# Deep exploration of logical models of cell differentiation in human preimplantation embryos

**DOI:** 10.1038/s41540-025-00537-7

**Published:** 2025-05-27

**Authors:** Mathieu Bolteau, Célia Messaoudi, Laurent David, Jérémie Bourdon, Carito Guziolowski

**Affiliations:** 1 Nantes Université, École Centrale Nantes, CNRS, LS2N, UMR 6004, F-44000 Nantes, France; 2 Nantes Université, CHU Nantes, INSERM, Center for Research in Transplantation and Translational Immunology, UMR 1064, F-44000 Nantes, France; 3 Nantes Université, CHU Nantes, CNRS, Inserm, BioCore, F-44000 Nantes, France

**Keywords:** Software, Developmental biology, Systems biology, Computer modelling, Computer science, Logic gates, Regulatory networks, Software

## Abstract

The advent of single-cell transcriptomics (scRNA-seq) has provided unprecedented access to specific cell type signatures, including during transient developmental stages. One key expectation is to be able to model gene regulatory networks (GRNs) from the cell-type scRNA-seq signatures. However, most computed GRNs are static models and lack the ability to predict the effects of genetic or environmental perturbations. Here, we focus on the maturation process of the trophectoderm (TE), the outer layer of cells of human embryos, which is critical for their ability to attach to the endometrium. Addressing this challenge required overcoming two major limitations: *(i)* handling the search space generated by the high dimensionality of single-cell data, *(ii)* the lack of feasible perturbation data for certain biological systems, which limits validation or generation of dynamic models. To address these challenges, we created SCIBORG, a computational package designed to infer Boolean networks (BNs) of gene regulation by integrating single-cell transcriptomic data with prior knowledge networks. SCIBORG uses logic programming to manage the combinatorial explosion. It learns two distinct BN families for each of the two developmental stages studied (TE and mature TE) by identifying specific gene regulatory mechanisms. The comparison between these two BN families reveals different pathways, identifying potential key genes critical for trophectoderm maturation. In silico validation through cell classification into studied stages reveals balanced precision 67% - 73% for inferred BN families. We demonstrate that SCIBORG is a powerful tool that integrates the diversity between gene expression profiles of cells at two different stages of development in the construction of Boolean models.

## Introduction

Understanding the regulatory mechanisms involved in the process of cell differentiation is central to the study of human embryonic development. The gradual acquisition of defined cellular functions governs human preimplantation development, ultimately leading to an implantation-competent embryo. Accessing transcriptomic profiles upon embryo development is currently the most powerful way to infer the regulatory events involved. Understanding human embryo development is expected to help to improve IVF success rates, which are limited to approximately 25%^1^. Given the limited possibilities to work on human embryos, we now need to build predictive models of human development, based on scRNA-seq datasets.

We distinguish two types of methods: the first one focuses on inferring gene regulatory networks (GRNs), while the second aims to model biological processes. GRNs, mathematically defined as graphs, are generally inferred de novo from gene expression measurements. GRN reconstruction methods provide reasonable estimates of gene connectivity but fail to capture regulatory interactions, such as activation or inhibition between genes. A commonly used approach based on gene correlation is SCENIC^2^, which uses scRNA-seq and ATAC-seq data, along with enrichment of the transcription factor binding motif, to infer signed and directed GRNs. However, these inference methods have limitations in predicting system behavior under genetic perturbations or environmental changes^3^. Addressing these challenges requires a deeper exploration of scRNA-seq data to enhance predictive capabilities. Several approaches have been proposed to infer mechanistic models from single-cell data measured at different stages of a cell differentiation process. Among these, approaches for deriving mechanistic logic models have gained particular attention. For example, Moignard et al.^4^ introduced SCNS, a toolkit for single-cell network synthesis, using single-cell expression of tens of well-characterized genes in early blood development to infer Boolean networks (BNs). These BNs allowed the researchers to identify regulatory mechanisms of 40 transcription factors that model cell differentiation in the system under study. However, SCNS does not incorporate prior knowledge networks and relies on a state transition graph, which requires high computational power to handle its combinatorial complexity. The work of Dunn et al.^5^, inferred mouse embryonic logic models from scRNA-seq and knockout data on mouse stem cells, revealing insightful results via a perturbation-based approach called Reasoning Engine for Interaction Networks (RE:IN). Meanwhile, Bonnafoux et al.^6^ developed WASABI, a method based on ordinary differential equations (ODEs) to infer a small erythroid differentiation regulatory network from single-cell temporal data. WASABI averages the signal to estimate protein and promoter activity levels, allowing the inference of dynamic regulatory interactions. Although this method incorporates proteomic and transcriptomic interactions, it cannot retrieve multiple regulations targeting a single species. Finally, a study by Chevalier et al.^7^ presents BoNesis which learned ensembles of dynamical logic models (dynamical BNs) associated with cell fate differentiation involved in tumor invasion and migration. This method processes single-cell data by averaging gene expression levels across differentiation stages. These four methods are complementary and antagonistic on certain criteria such as the search of regulatory mechanisms, the exhaustivity of the search space explored, the type of model they provide, and the inclusion of the heterogeneity of single cell gene expression profiles. In Supplementary Note 1, we present a more in-depth comparison of these methods according to these criteria and position SCIBORG.

To fix the above issues, our study addresses scenarios where experimental perturbations are not feasible due to ethical, biological, or legal constraints. In addition, our method emphasizes capturing the heterogeneity of gene expression data at each stage of cell differentiation, thereby maximizing the diversity of expression profiles. In our approach, we set aside the dynamic aspects of the models and introduce an optimization constraint focused on maximizing the heterogeneity of expression profiles. This strategy narrows the search space, allowing for better convergence, while additional constraints effectively mitigate the combinatorial explosion inherent in the problem. This work is an improvement of the tool presented in Bolteau et al.^8^, which inferred static BNs (as opposite to dynamical BNs) using answer set programming (ASP). Here, we present SCIBORG, a system that significantly improves computational efficiency by drastically reducing both memory usage and execution time (from 65h to 7h) while also processing larger datasets. These results in a more detailed exploration of outcomes, producing exhaustive and robust results.

Focusing on trophectoderm (TE) and mature TE developmental stages, SCIBORG used single-cell transcriptomic data and incorporated prior knowledge from databases, along with identified pseudo-perturbations, to infer stage-specific families of Boolean networks. We validated the inferred models, confirming their ability to identify regulatory mechanisms specific to different developmental stages. This advance not only overcomes previous computational challenges but also establishes SCIBORG as a powerful tool for studying complex gene regulatory processes in developmental biology. Moreover, the flexibility of our approach allows for its extension to other developmental stages and a wide range of biological studies. A similar analysis showing the adaptability of SCIBORG to other developmental stages can be found in Supplementary Note 2.

## Results

### SCIBORG infers BNs to model two developmental stages

This study was conducted using scRNA-seq data collected from human embryos. The dataset, refined in Meistermann et al.^9^, contains the expression profiles of 34, 054 genes in 1496 cells (see Methods). Considering a cellular differentiation phenomenon, such as human embryonic development, we postulate that a cell at a specific stage can either remain in that stage or differentiate into a next stage.

**Fig. 1:**
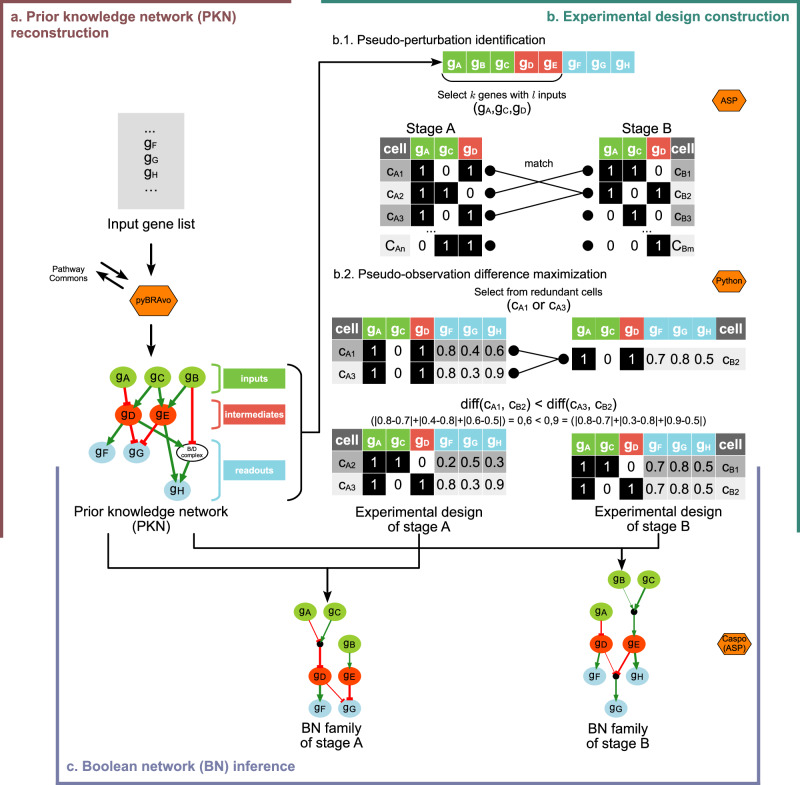
SCIBORG overview. **a** The prior knowledge network (PKN) reconstruction is performed using the pyBRAvo tool. Starting from an input gene list, pyBRAvo queries the Pathway Commons database to identify predecessor genes, thereby generating a signed and oriented graph. The PKN consists of four node types: input genes (green, nodes without predecessors), readout genes (blue, nodes without successors), intermediate genes (red, other genes), and protein complexes (white). **b** Stage-specific experimental designs are constructed using an answer set programming (ASP) program to identify pseudo-perturbations based on parameters *k* and *l*. In this example, with *k* = 3 and *l* = 2, the selected genes are *g*_*A*_, *g*_*C*_, and *g*_*D*_. A maximum of two pseudo-perturbations are identified, with two redundant cells, *c*_*A*1_ and *c*_*A*3_. The redundant cells involved in pseudo-perturbations are chosen by maximizing the pseudo-perturbation difference (implemented in Python). In this example, cell *c*_*A*3_ is selected, resulting in the formation of the two stage-specific experimental designs shown. **c** Using the reconstructed PKN and the stage-specific experimental design, the tool Caspo (based on ASP) is used to infer a family of Boolean networks (BNs) for each studied stage.

The central challenge is to identify the logical mechanisms, represented as Boolean networks (BNs), that govern cellular behavior at the initial developmental stage and those that regulate the final stage. Following the approach of previous studies^4–7^, we assume that cellular behavior is driven by logical rules underlying gene regulatory networks. In such networks, the signal flows from a set of so-called entry-genes to a set of output-genes. Identifying these logical rules often requires perturbation experiments that track how gene states evolve from an initial expression profile (under perturbation) to a final one. However, due to ethical and legal constraints, performing perturbation experiments is not feasible in certain contexts, including human embryonic studies. To address this limitation, we introduce the concept of *pseudo-perturbations*, derived from single-cell transcriptomic data. Pseudo-perturbations mimic perturbations by identifying specific gene expression patterns that are consistent across cells at different developmental stages. These correspond to groups of cells where the same set of entry-genes exhibits similar expression profiles in both developmental stages (see Fig. 1b.1). By analyzing the similarities and differences in the expression of entry-genes and their corresponding output-genes within the same cells, we can generate hypotheses about the logical mechanisms regulating gene networks.

For clarity, we define the terminology used throughout this paper. A pseudo-perturbations refer to a set of gene observations, across a subset of cells, having the same expression in both developmental stages, as shown in Fig. 1b.1. A single pseudo-perturbation compares two cells at the initial and final developmental stages. *Pseudo-observations* refer to the output-gene observations within the cells selected for the pseudo-perturbations, as shown in Fig. 1b.2. Thus, pseudo-perturbations and pseudo-observations, extracted from single-cell data, will be used to mimic a perturbation-like scenario to model the cellular behavior in form of BNs at the initial and final stages.

To discover GRN changes upon maturation of trophectoderm, SCIBORG integrates possible gene expressions as pseudo-perturbations and pseudo-observations to infer BNs modeling the two developmental stages under study. SCIBORG operates in three steps (Fig. 1): *(i)* prior knowledge network (PKN) reconstruction, *(ii)* experimental design construction, and *(iii)* BN inference.

First, the PKN defines the domain of gene interactions to consider for modeling. The PKN is reconstructed using an input gene list through queries on databases using pyBRAvo tool integrated into SCIBORG (Fig. 1a, Methods). The resulting PKN is a directed and signed graph where nodes represent gene or protein-complexes, and edges represent interactions (activation or inhibition) between these entities. Within the PKN, we identify three types of genes according to the graph topology: input, intermediate and readout genes. Input and intermediate genes form the basis for the search of the pseudo-perturbations, while pseudo-observations are derived from readout gene expression profiles (Fig. 1a).

Second, the experimental design construction involves identifying pseudo-perturbations and maximizing the differences in their associated pseudo-observations (Fig. 1b). Considering a set of *k* binarized input-intermediate genes, we use an ASP program to maximize the number of pseudo-perturbations defined as pairs of cells with identical *k*-gene expressions, but coming from two different stages (Fig. 1b, Methods). This ASP program is issued from successive improvements of an initial program presented in Chebouba et al.^10^, which was adapted to fit with our case study and be more efficient (see Methods for details). Multiple cells in a stage can have the same *k*-gene expression, referring to redundant cells (see Methods for details). Among all redundant cells, we maximize the pseudo-observation differences between the two stages to distinguish the stages as much as possible. Differences between normalized readout gene expressions are computed pairwise, and the cell pair yielding the highest difference is selected (Fig. 1b, Methods). Stage-specific experimental designs (Fig. 1b) are obtained by combining pseudo-perturbations and their associated pseudo-observations.

Third, the reconstructed PKN and the experimental designs serve as inputs for inferring BNs that model each stage using the Caspo tool^11^ integrated into the SCIBORG software (Fig. 1c, Methods). Briefly, Caspo employs ASP to identify the smallest BNs that satisfy the constraints imposed by the PKN and align with the gene expression profiles defined in the experimental designs (Methods). These inferred families of BNs model the regulatory mechanisms underlying the two studied stages through logical gates that govern gene interactions (Fig. 1c).

SCIBORG builds upon and enhances the method introduced in Bolteau et al.^8^. First, we reconstruct a larger PKN than the one used in the previous study (see Methods), enabling the inclusion of a greater number of gene interactions as prior knowledge. Second, we refine the data preprocessing by modifying the normalization of readout genes to enhance the robustness of downstream analyses (see Methods). The most significant improvement, however, lies in the logic programming framework used for pseudo-perturbation identification. We reformulate key logical rules to optimize both computational memory usage and processing time (see Methods; Supplementary Table 1; Supplementary Note 5). These refinements allow SCIBORG to efficiently handle larger PKNs and identify a greater number of pseudo-perturbations within reduced computational time. As a result, the method moves closer to optimality in pseudo-perturbation identification, offering a more scalable and efficient approach.

### SCIBORG allows for exhaustiveness of the identified pseudo-perturbations

**Fig. 2:**
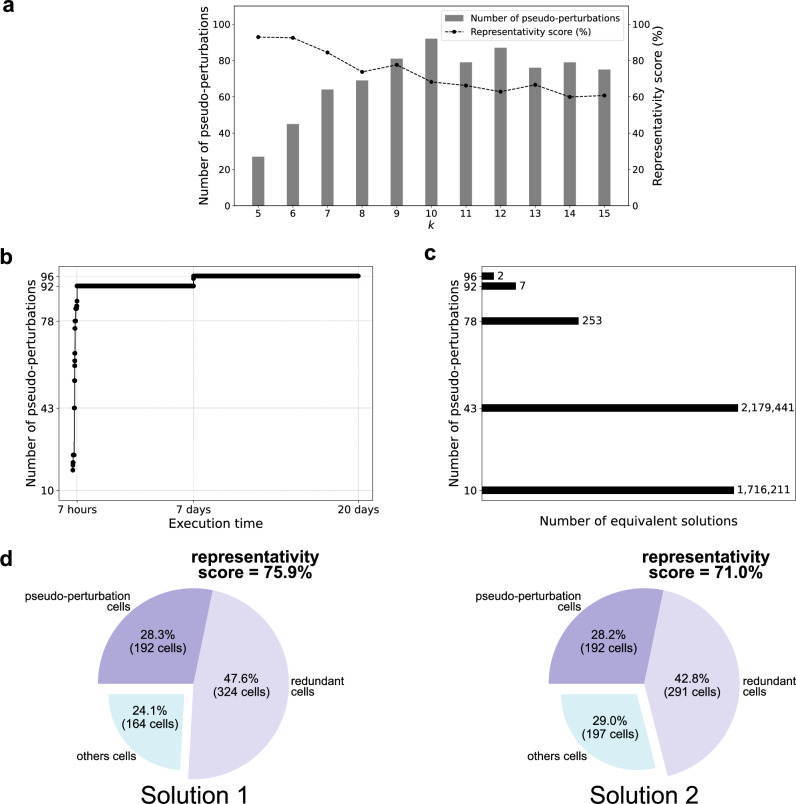
Convergence and exhaustiveness of pseudo-perturbations solutions. **a** Exploration of different values for the parameter *k* for the pseudo-perturbation identification program. The number of pseudo-perturbations obtained after 30 hours of computation on a computer cluster are plotted with their representativity score. **b** Convergence of the identified pseudo-perturbations over time. The results were obtained using a computer cluster. **c** Equivalent solutions for a subset of key points (10, 43, 78, 92 and 96 pseudo-perturbations). The number of equivalent solutions is sub-optimal because the search was stopped after 7 days of computation on a computer cluster. **d** Representativity score of the two equivalent solutions of the key point 96 pseudo-perturbations.

Given the pseudo-perturbation identification program, we explored different values of *k*-selected genes going from 5 to 15 to identify the best value maximizing the number of pseudo-perturbations. This analysis was run and intentionally interrupted at 30 hours (Fig. 2a). We can observe that the number of pseudo-perturbations fluctuates with *k*, reaching a maximal value of 92 pseudo-perturbations for *k* = 10. In addition, we calculated the cell representativity for each test (see Methods), defined as the proportion of selected cells relative to the total cell population for a given stage. This metric informs on the performance of the chosen pseudo-perturbations to represent the broader cell population. Overall, cell representativity decreases as *k* increases. By selecting more experimental single-cell data, Caspo can better identify discriminatory regulatory mechanisms. Notice, for example, that when we learned BNs using the 75 pseudo-perturbations identified for *k* = 15 (see Supplementary Fig. 1). The resulting BNs are smaller in terms of logical rules and exhibit lower precision, as indicated by the MSE (see Methods for definition). These results reinforce the necessity of finding a compromise between the number of pseudo-perturbations and the choice of *k*; we thus selected *k* = 10 for the rest of our analyses.

After setting the number of considered genes, we monitor the number of pseudo-perturbations identified by the program over time (Fig. 2b). As we stopped the program after 20 days, the pseudo-perturbations found are sub-optimal solutions. We observed an exponential increase in the number of identified pseudo-perturbations until a plateau was reached, with 96 identified pseudo-perturbations (consistent from day 7 to day 20). This convergence suggests that our results are probably close to optimality. We then compared the nature of pseudo-perturbations identified relative to the number of iterations. Looking at 10, 43, 78, 92, and 96 pseudo-perturbations, we identified a convergence in the number of equivalent solutions over time (see Fig. 2c). Interestingly, there is a significant disparity in the number of solutions when comparing key point 10 (1, 716, 211 solutions) to key point 43 (2, 179, 441 solutions). A larger number of equivalent solutions was observed at the intermediate key point (43), rather than the earlier stage (10). This can be attributed to the sub-optimal nature of the calculations, which were halted after a pre-determined 7-day timeout. For the 96 pseudo-perturbations plateau, 2 equivalent subsets of *k* = 10 genes were observed. The stark contrast between the large number of equivalent solutions (exceeding 1 million) in the initial stages of pseudo-perturbation identification and the minimal number of solutions at the plateau could be explained by data sparsity. For example, given the high number of zeros in the dataset, the likelihood of finding 10 cells where a gene is not expressed is relatively high. In contrast, identifying 96 cells (out of 300) with similar non-expression patterns is far less probable. Consequently, the number of equivalent solutions selecting different sets of genes could grow exponentially when considering few pseudo-perturbations.

We then compared the cell representativity score (see Methods) of the 96 pseudo-perturbations identified by the two solutions. The first solution had a score of 75.9%, while the second one of 71%. These scores indicate that the pseudo-perturbation set from solution 1 fits with the expression of the 10 selected genes in 75.9% of the total cell population across both stages, while the genes are present in 71% of cells for solution 2. To contextualize these findings, we divided the total cell population into three subsets: *(i)* the 192 cells (96 of each stage) corresponding to the selected pseudo-perturbations, *(ii)* the redundant cells, defined as cells with gene expression profiles similar to the pseudo-perturbations, and *(iii)* cells without similarity in the gene expression profile with respect to the pseudo-perturbations (Fig. 2d). Additionally, the representativity scores obtained for the two solutions at the plateau (75.9% and 71%) are notably higher than the score observed during the *k* parameter exploration for 92 pseudo-perturbations (68.2%; see Fig. 2a, *k* = 10). Using cell representativity as a criterion to select between equivalent solutions, solution 1 would be the preferred choice.

In summary, we select 10 genes and identify 96 pseudo-perturbations in each developmental stage. The selected genes and the cells involved in pseudo-perturbations were exhaustive with respect to the equivalent solutions that could have been obtained. Notably, as the number of identified pseudo-perturbations increased, the number of equivalent solutions decreased drastically. Moreover, for each solution, the 96 cells involved in pseudo-perturbations represented more than 70% of the total cell population, further emphasizing the exhaustiveness of our approach.

### SCIBORG explores the robustness through the learned models

We explore the robustness of our models using two complementary approaches. First, we analyzed the gene composition of equivalent solutions. By equivalent solution we mean a different set of *k* genes that allows us to obtain the same number of pseudo-perturbations. Second, we evaluated the performance of the BN models, trained on pseudo-perturbation data, in classifying the remaining cells.

**Fig. 3:**
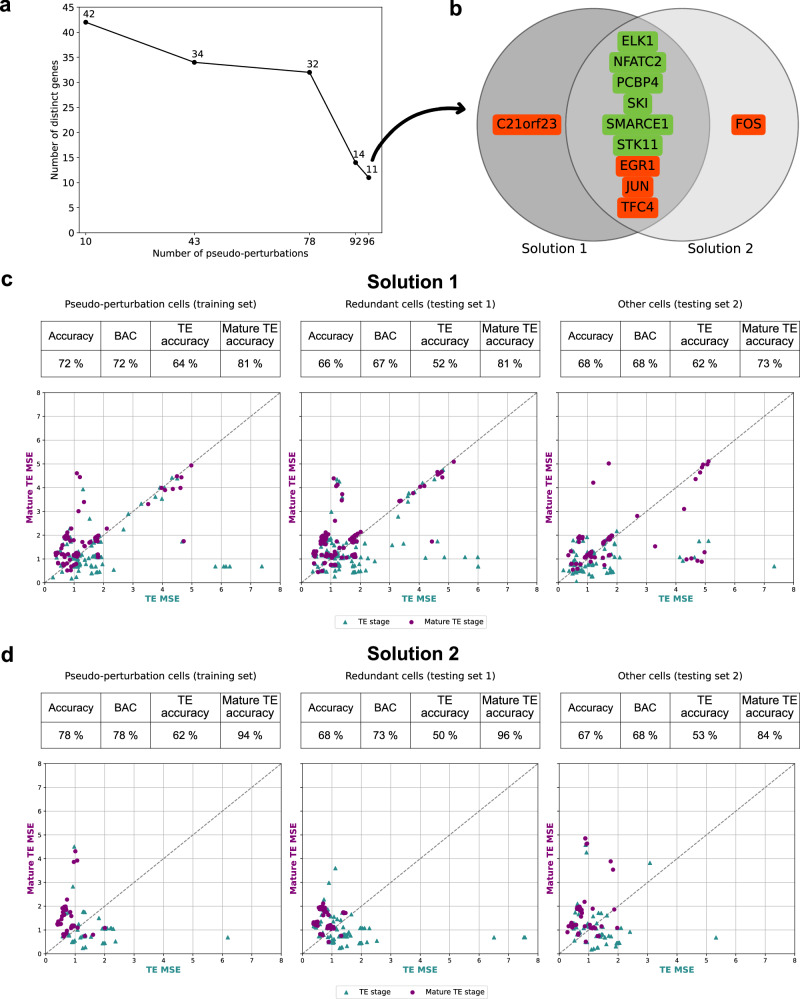
Robustness of the equivalent solutions and learned BN models. **a** Exploration of distinct genes composing the equivalent solutions for a subset of key points. **b** Gene composition of the two equivalent solutions for the key point 96 pseudo-perturbations. **c** Cell classifier results for solution 1. Negatively log-transformed MSE values. **d** Cell classifier results for solution 2.

As observed earlier, different sets of *k* genes can yield equivalent solutions, where the same number of cells is selected. To examine the consistency of gene selection, we calculated the number of distinct genes across all equivalent solutions (Fig. 3a). We observe that the number of distinct genes is significantly smaller than the total number of considered genes (121). Moreover, this number decreased as the number of identified pseudo-perturbations increased. For instance, among the 2 million solutions found at key point 43, only 34 unique genes consistently appeared in the subsets of *k* = 10 genes. For key point 96, the two solutions shared 9 out of 10 genes, differing by only a single gene: *C21orf23* in one solution and *FOS* in the other (Fig. 3b). This analysis suggests that, despite the small number of equivalent solutions, both exhibit robustness in terms of their gene composition.

In this study, we introduce a method named the *cell classifier*, which, given two BN families representing two developmental stages, classifies a new incoming cell into a specific stage. This method uses a mean square error (MSE) metric to compare the stage-specific BN family with the expression data of the incoming cell, assigning it to the family with the lowest MSE (see Methods for details). We applied this method to the two equivalent solutions, namely solution 1 and 2, obtained for the key point 96. The purpose was to evaluate whether the inferred BN families can distinguish between developmental stages when applied to new expression data. We considered the same three sets of cells as in the previous section: the pseudo-perturbation cells, which serve as the training set since they were used to learn the stage-specific BN families, and two testing sets composed of redundant cells and other cells, respectively.

**Fig. 4:**
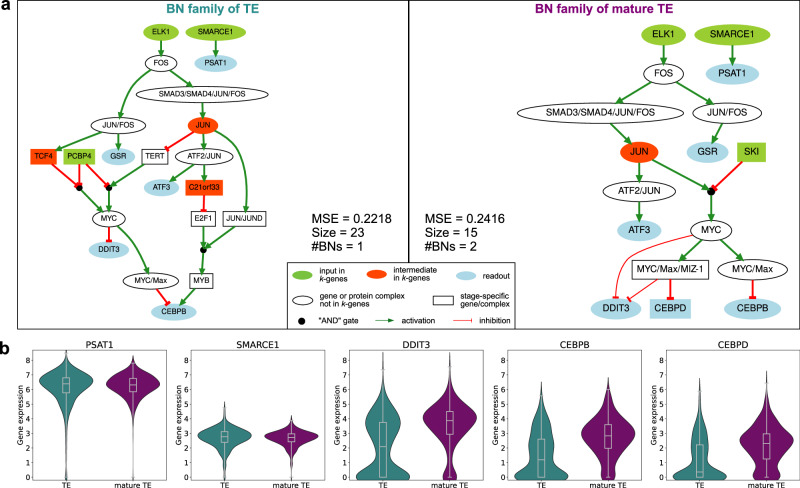
Learned BNs and gene expression profiles. **a** Families of learned BNs for TE and mature TE stages. Each network represents the union of (sub-)optimal BNs learned from the reduced PKN and the experimental design. The colored nodes represent genes associated with experimental designs, including input and intermediates involved in pseudo-perturbations, and readout genes involved in pseudo-observations. The width of the arc represents the frequency of occurrence of this interaction in the BNs. We set the following parameter values for the BN learning: *fitness*_*tolerance* = 0.0001, *size*_*tolerance* = 0 and *length* = 2. **b** Expression profiles for a subset of genes included in the BNs across all cells. Log-transformed gene expressions (see Methods).

Our results show that the training set exhibits a higher balanced accuracy (BAC) score compared to the testing sets, with values of 72% vs. 67%/68% for solution 1 (Fig. 3c, d). Similar results are obtained for solution 2: 78% vs. 73%/68%. These findings are consistent with the expectation that models typically perform better with the data they were trained on than with unseen data. In both solutions, mature TE BNs are more accurate compared to TE ones, regarding stage-specific accuracy. The inferred BNs, used for this cell classifier analysis, are presented in Fig. 4 for solution 1 and in Supplementary Fig. 2 for solution 2.

Interestingly, the models from solution 1 demonstrate a stronger ability to discriminate between the two stages compared to those from solution 2. In solution 2, BN models fail to classify 245 cells (out of 680) because the MSE scores for both stages are indistinguishable. In contrast, solution 1’s BN models successfully assign a stage to every cell. Overall, these results indicate that SCIBORG enables the learning of accurate BNs, particularly for the BN models obtained from solution 1, and provides metrics to guide the solution choice.

We further examine the predictions for each individual cell in more detail. In Fig. 3c, d, we represent the calculated MSEs through TE BNs versus the calculated MSEs through mature TE BNs for each cell within the three sets of cells: training set, testing set 1 and testing set 2. For each set, we represent all cells where a stage-classification is possible across both stages (TE and mature TE) in function of their TE and mature TE MSEs. If the cell lies below the dashed line, it is classified in the TE group; otherwise, it is classified as belonging to the mature TE group. This plot allows us to visually assess the correctness of classification based on each cell’s position. Since the MSE values are negatively log-transformed (see Methods), higher values correspond to stronger confidence in classification, while lower values indicate lower confidence. Considering the training set for both solutions, we observe that most misclassified cells from both stages are located near the dashed line, suggesting that only minor adjustments would be needed to correct these classifications. For solution 1, errors appear along the diagonal and also in areas where both stage-BNs exhibit high MSE values. Notably, there are misclassified mature cells that show a low MSE (high inverse MSE value) for TE models, but a high MSE (low inverse MSE value) for mature TE models. This discrepancy suggests that either the cell annotation was incorrect or the BN models were poorly inferred for these cells. These mislabeled cells represent an intriguing direction for future investigation.

Furthermore, we analyze the performance of the cell classifier while applying a threshold on the MSE to exclude ambiguous cases (see Methods). Specifically, we exclude cells for which the MSE values are similar in the two studied stages, as they do not contribute meaningfully to classification. This approach improves classification accuracy from 69% to 72% in BN-families for solution 1, using a threshold interval of [*MSE* − 0.01, *MSE* + 0.01] (espilon 0.01; Supplementary Fig. 3). In this setting, 65 out of 680 cells remain unclassified.

Based on these results, solution 1 emerges as the most reliable model. It exhibits correct overall accuracies with no unclassified cells, indicating that the BNs inferred in solution 1 more effectively model the cells, even when there are significant differences between the training and testing sets.

### SCIBORG identifies regulatory mechanisms and potential marker genes in the learned models

Given that the cell representativity and classifier accuracy for solution 1 are higher, we next analyze the BN families learned (Fig. 4a). Notably, TE requires more interactions to model, as indicated by the greater number of logical gates (23 vs. 15). These interactions are essential because Caspo learns only one BN for TE, while it learns two distinct BNs for mature TE. The two mature TE BNs differ primarily in the inhibition of *DDIT3*, that can be mediated by *MYC* or through the *MYC/Max/MIZ-1* complex. We provide the logical rules that make up the learned BNs in Supplementary Note 6. The learning error regarding the MSE is higher for mature TE (0.2416) than TE (0.2215), suggesting that mature TE is more complex to model using the gene expression data from the 96 cells.

The TE BN consists of 3 inputs and 3 intermediates, whereas the mature TE BNs include 3 inputs and only 1 intermediate. Additionally, the mature TE model incorporates an extra readout gene, *CEBPD*, which is absent in the TE model. Despite these differences, similar interactions are observed in both models, such as the activation of *PSAT1* by *SMARCE1*. When examining the expression profiles of these two genes (Fig. 4b), we find that both are highly and similarly expressed at both stages, suggesting a potential correlation. This finding is further corroborated by their activation in our learned models. For a comprehensive view, we provide the expression profiles of all genes included in the learned BNs in Supplementary Fig. 4.

Our stage-specific BNs reveal distinct regulatory mechanisms. Notably, in TE, *CEBPB* is regulated via multiple pathways, including through *SMAD3/SMAD4/JUN/FOS* and *MYB*, or through *JUN/FOS* and *MYC*. In contrast, in mature TE, *CEBPB* is regulated by a single pathway through *SMAD3/SMAD4/JUN/FOS* and *MYC*. These mechanisms, suggested by our BNs, provide an explanation for the differences observed in *CEBPB*’s expression profile, which varies between the TE and mature TE stages (Fig. 4b).

We notice similar expression profiles for the genes *DDIT3* and *CEBPD* with respect to the two stages studied. Both are highly expressed in most cells in mature TE and less expressed in TE cells. Although not identical, these expression patterns indicate that *DDIT3* and *CEBPD* guide the cell in the same direction. In comparison to the marker of mature TE, *NR2F2*^9^, we observe similar expression (see Supplementary Fig. 5). These three genes seems correlated to *NR2F2*. Further experimental explorations should be performed to confirm this hypothesis.

In summary, SCIBORG enables the identification of distinct regulatory mechanisms that differentiate the developmental stages under study. Moreover, it highlights potential key genes involved in the maturation of the TE, offering insights into their possible roles in early developmental processes.

## Discussion

In this study, we present SCIBORG, an innovative computational method to generate BNs from scRNA-seq data during cell fate transitions. As a proof of concept, we studied the maturation process of trophectoderm (TE) in preimplantation embryos.

Compared to state-of-the-art methods based on Boolean formalism, SCIBORG integrates gene expression data from a maximum number of cells, enabling the identification of specific behaviors critical to cell differentiation. Furthermore, our framework exhaustively enumerates models through convergence, providing increased confidence and robustness in the identified regulatory mechanisms. This approach complements other computational techniques^4,5^ that often rely on system perturbations to validate models. Instead, we propose an in silico validation using a cell classifier. SCIBORG is also designed to be highly adaptable. For example, the PKN reconstruction step is optional, allowing users with an already well-defined PKN to seamlessly integrate it and proceed with the subsequent steps of the SCIBORG pipeline.

We applied SCIBORG to identify the gene regulatory mechanisms that distinguish TE and mature TE stages. From the full dataset comprising 680 cells and 121 genes, we identified 10 genes whose expression profiles were observed across 96 cells in each of the TE and mature TE stages, which we term *pseudo-perturbations*. The complexity of such calculation for the worst-case is $$\left(\begin{array}{c}121\\ 10\end{array}\right)\times {2}^{348\times 332}=8.01\times {10}^{34793}$$(see Methods; Equation (2)). Our program outperforms existing techniques in previous studies^8,10,12^ in terms of the number of identified pseudo-perturbations, as well as execution time and storage efficiency (see Supplementary Table 1). The combination of pseudo-perturbations and pseudo-observations provides input-output gene expression behaviors that are used to learn Boolean models. SCIBORG enables the enumeration of two solutions for model learning, allowing us to infer families of Boolean networks (BNs) that capture gene regulatory mechanisms specific to TE and mature TE stages. Our results suggest that *CEBPB*, *CEPBD* and *DDIT3* genes play critical roles in TE maturation. Through the Boolean models, we identify the gene orchestration involved in these stages. Additionally, the model validation process indicates that SCIBORG learns accurate Boolean models of both developmental stages; BNs learned from 192 cells are 67–73% (see Fig. 3c, d) accurate with respect to the ones not selected in the experimental design construction step (488 cells).

This work is open to various perspectives, either on the PKN reconstruction or on the BN inference. We could imagine applying different methods to enhance the quality of the reconstructed PKN. First, by using an already available PKN characteristic of the biological system studied. Second, a refinement of the PKN reconstructed by our method could be done by including other genetic information such as TF-target enrichment coming from methods such as SCENIC. Another perspective would be to combine the families of Boolean Networks inferred by multiple equivalent solutions and quantify if this combination proposes better stages discrimination.

In SCIBORG, pseudo-perturbations play a key role in inferring BN families, acting as substitutes for real perturbations, which are often inaccessible due to the aforementioned concerns. These pseudo-perturbations are currently derived from scRNA-seq data. Recently, in ref. ^13^, the authors derive real perturbations that highlight the pivotal role of glucocorticoid exposure during the preimplantation stage of human embryos. This development opens the door to comparing real and pseudo-perturbations within a controled experimental framework.

We anticipate that SCIBORG will be a valuable tool for discovering key mechanisms when applied to other developmental stages of human embryogenesis. Furthermore, SCIBORG will be a versatile tool for modeling a wide range of biological systems. Looking forward, our future objective is to integrate dynamic aspects of the studied systems, enabling the modeling of entire cell differentiation processes and further expanding SCIBORG’s applicability to complex biological research.

## Methods

### Data

Throughout this study, we exploit scRNA-seq data obtained from stage-matched human embryos, leveraging the dataset initially compiled by Petropoulos et al.^14^ and subsequently refined in Meistermann et al.^9^. This dataset encompasses the expression profiles of 34, 054 genes across 1, 496 cells derived from 88 stage-matched human embryos. We consider the count matrix as base of our data, encompassing raw data.

We create diverse toy datasets from single-cell data for method testing (Table 1). Considering TE and mature TE, we define the dataset *SC* characterizing our case study. In addition, we consider also a phosphoproteomic dataset (*P*), from Chebouba et al.^10^, for testing and comparing our different pseudo-perturbation identification programs.

**Table 1:** Datasets description

Dataset	Source	Class name(C1;C2)	Genes^a^	Cells^b^	#C1’s cells^b^	#C2’s cells^b^
A	Artificial	C1;C2	10	10	5	5
B	Subset of single-cell data	*E*^*TE*^; *M*^*TE*^	30	24	12	12
C	Subset of single-cell data	*E*^*TE*^; *M*^*TE*^	100	50	25	25
D	Subset of single-cell data	*E*^*TE*^; *M*^*TE*^	120	200	100	100
SC	Single-cell data	*M*^*TE*^; *L*^*TE*^	111	680	348	332
P	Phosphoproteomics data^c^	CR ; PR	79	191	136	55

*E*^*TE*^ early TE, *M*^*TE*^ TE, *L*^*TE*^ mature TE, *CR* Complete Remission, *PR* Primary Resistant (see ref. ^10^).

^a^For dataset P, proteins are studied (not genes).

^b^For dataset P, patients are studied (not cells).

^c^From ref. ^10^.

The artifical dataset (A) consists of binarized gene expression values randomly generated with 0 and 1 for 10 genes across 10 cells. Datasets B, C and D, are subsets of single-cell dataset, created by randomly selecting a specified number of cells. The goal was to construct datasets of increasing size, both in terms of the number of cells and genes.

### Data preprocessing

We first select cells involved in the studied stages, particularly TE and mature TE, representing 348 and 332 cells, respectively. Input and intermediate genes (see next section) in these cells are binarized respecting the following rule: if 2 reads or more are expressed, we consider the gene active (represented by a 1), otherwise the gene is inactive (represented by a 0). Readout genes (see next section) are normalized between 0 and 1 following the equation:

1 $${n}_{ij}=\frac{2}{\pi }\times \arctan ({r}_{ij})$$

where, *n*_*ij*_ represents the normalized expression of the gene *j* for the cell *i* and *r*_*ij*_ denotes the raw expression of the gene *j* for the cell *i*.

### PKN reconstruction

To reconstruct the PKN, which forms the basis of prior knowledge to infer BNs, we use the pyBRAvo tool^15^, integrated into SCIBORG. Given a list of genes, pyBRAvo makes recursive queries on the (locally hosted) Pathway Commons database^16^ to find predecessors of the input genes. This process continues until a user-defined depth is reached or the largest possible PKN is reconstructed. For further details on the queries made and Pathway Commons hosting, refer to Supplementary Note 3.

Based on the network topology, we define three types of genes: *(i)* input genes, which have no predecessors (represented in green in Supplementary Fig. 6) ; *(ii)* readouts, which have no successors (represented in blue) ; and *(iii)* intermediates, which are the other genes (represented in orange). The reconstructed PKN is then reduced in order to include only genes present in the expression matrix. Protein-complexes between these genes are also retained to maintain as much connectivity as possible. In addition, input genes directly connected to readout genes, without any intermediates or protein-complexes are removed to focus on regulatory mechanisms involving various types of genes. This choice is done to avoid direct connections which could be trivially found using the discretized data, and therefore induce the logic program solver to propose trivial topologies of BNs.

The PKN presented in Supplementary Fig. 6, including gene-transcriptional events, was reconstructed using the regulatory network reconstruction capabilities of pyBRAvo, leveraging Pathway Commons v13, and excluding miRTarBase, MSigDB, and CTD databases to eliminate miRNA and toxicogenomics interactions. An infinite depth was set to reconstruct the largest possible PKN from the 438 transcription factors identified as relevant in human embryonic development^9^ provided as input (gene list in Supplementary Note 4). In addition, we enabled the use of synonyms, complex decomposition, and suffix label expansion, three parameters of pyBRAvo (see ref. ^15^ for more details). The reconstructed PKN, after reduction following the expression matrix (Supplementary Fig. 6), comprises 369 edges and 225 nodes, including 85 inputs, 36 intermediates and 19 readouts. The other nodes correspond to protein-complexes. Of the 438 initial TFs provided as input, only 28 were found in the PKN.

### Experimental design construction

An experimental design is specific to a studied stage and composed of two parts: *(i)* the pseudo-perturbations, which consist of binarized values for certain cells for *k* selected genes, *(ii)* the pseudo-observations, which are the normalized values of the readout genes of the cells involved in the pseudo-perturbations. To construct the experimental design, we first identify the pseudo-perturbations and then maximize the pseudo-observation differences.

#### Pseudo-perturbation identification

The pseudo-perturbation identification process is launched using answer set programming (ASP) paradigm^17^, suited for solving combinatorial problems. The program used in SCIBORG is an improvement in terms of computational memory and time of the program presented in our previous works^8,12^.

Hereafter, we define the problem statement of the pseudo-perturbation identification process. Let us define a binary matrix *E*, where *e*_*ij*_ represents the presence or absence of gene *j* for cell *i*. We denote by *C* the set of cells and by *G* the set of input and intermediate genes identified in the previously reconstructed PKN. Each cell in *C* is associated with one developmental stage, either *A* or *B*; thus *C* = *A*⊎*B*. We define the relation *I*^*G*^, *I*^*G*^(*c*_*i*_) = {*g*_*j*_ ∈ *G*∣*e*_*ij*_ = 1}, where *I*^*G*^(*c*_*i*_) represents the active genes, belonging to *G*, for cell *c*_*i*_. If $G^{\prime} \subset G$, then the restriction of *I*^*G*^ to $G^{\prime}$ is defined by ${I}^{G^{\prime} }({c}_{i})={I}^{G}({c}_{i})\cap G^{\prime}$. Let us define the function type_of(*g*_*i*_), which provides the type of the gene *g*_*i*_ that can be: ‘input’ or ‘intermediate’. We define also the function ancestor(*g*_*i*_, *g*_*j*_), meaning that a link exists from the *g*_*i*_ to the gene *g*_*j*_, regarding the network topology of the PKN.

Given a matrix *E*, a parameter *k* limiting the number of selected genes, and a parameter *l* giving the proportion of input genes in the *k* selected genes, find a subset $G^{\prime}$ of genes and the largest subset ${C}^{{\prime} }$ (${C}^{{\prime} }={A}^{{\prime} }\uplus {B}^{{\prime} }\subset C$, where ${A}^{{\prime} }\subset A$ and ${B}^{{\prime} }\subset B$) satisfying the following four constraints:

- *Constraint 1*. The size of ${G}^{{\prime} }$ is fixed to *k*, where $| \left\{{g}_{i}\in {G}^{{\prime} },\,{\text{type}}{\_}{\text{of}}({g}_{i})= {\mbox{`}} {\text{input}}{\mbox{'}} \right\}| =l$, with ${g}_{i}\in {G}^{{\prime} }$.
- *Constraint 2*. $\forall {c}_{1},{c}_{2}\,\in {A}^{{\prime} }$ (resp. ${B}^{{\prime} }$), *c*_1_ ≠ *c*_2_, we ensure that ${I}^{{G}^{{\prime} }}({c}_{1})\,\ne\, {I}^{{G}^{{\prime} }}({c}_{2})$.
- *Constraint 3*. $\forall {c}_{1}\,\in {A}^{{\prime} }$ (resp. ${B}^{{\prime} }$), $\exists {c}_{2}\in {B}^{{\prime} }$ (resp. ${A}^{{\prime} }$), we ensure that ${I}^{{G}^{{\prime} }}({c}_{1})={I}^{{G}^{{\prime} }}({c}_{2})$.
- *Constraint 4*. ∀ *g*_1_, with type_of(*g*_1_) = ‘intermediate’, ∃ *g*_2_, with type_of(*g*_2_) = ‘input’, such that ancestor(*g*_2_, *g*_1_).

The constraint 1 reduces the search space to improve the computational efficiency. The program selects *k* genes, we ensure that a proportion *l* input genes from the *k* selected genes, where *l* < *k*. The remaining genes (*k* − *l*) will be intermediates. This ensures that both input and intermediate genes are included in the *k* selected genes. The constraint 2 prevents redundancy within a same class by forbidding cells from having the same gene expression for the *k* selected genes. The constraint 3 ensures a match between two identified cells from different class, having the same gene expression for the *k* selected genes. The constraint 4 ensures connectivity between inputs and intermediates. We add this constraint in order to have set of *k* genes that are connected to each other and potentially yielding the inference of input-intermediate-readout connected BNs.

We refer as *pseudo-perturbations* to the optimal assignment ${G}^{{\prime} }\times {C}^{{\prime} }$ maximizing $| {C}^{{\prime} }|$. Multiple sets of *k* genes can lead to the same number of pseudo-perturbations. We refer to these outcomes as equivalent solutions. In our study, we explore these equivalent solutions by fixing the number of pseudo-perturbations to be found and enumerating the possible *k*-gene sets that lead to the fixed pseudo-perturbation number.

The ASP program identifying pseudo-perturbations is a more efficient version of a previous program presented in refs. ^8,12^. We modify the rules concerning the Constraint 4 (see Results) by transforming rules by constraints in order to reduce the search space and thus improve the efficiency of the program in terms of computation storage and time. Compared to state-of-the-art programs on different datasets, SCIBORG’s program we present in this study enables the identification of a larger number of pseudo-perturbations in drastically less execution time (see Supplementary Table 1). For instance, in our case study, we identify 92 pseudo-perturbations in 7 hours compared to 20 pseudo-perturbations in 65 hours. For further details on the ASP program with a line-by-line explanation, please refer to Supplementary Note 5.

The complexity of this program can be analyzed based on the selection of *k* genes from a total set of *G* genes and the pairing of cells. For each possible subset of *k* genes, a total of *c* associations between cells in classes *A* and *B* (where the values of the *k* genes match) must be tested to eliminate redundancies within the same class. The maximum possible value of *c* is ∣*A*∣ × ∣*B*∣, corresponding to the case where all cells from both classes are paired. The solver employs backjumping and conflict-driven learning to optimize the search space, meaning our estimate represents a worst-case scenario. The estimated complexity (see Equation (2)) suggests that our algorithm has an exponential complexity with respect to the number of selected genes and the number of cells in the scRNA-seq dataset:

2 $${\mathcal{O}}\left(\left(\begin{array}{c}| G| \\ k\end{array}\right)\times {2}^{| A| \times | B| }\right)$$

where ∣*G*∣ is the total number of genes, *k* is the number of selected genes, and ∣*A*∣ (resp. ∣*B*∣) denotes the number of cells in class *A* (resp. class *B*).

#### Pseudo-observation difference maximization

Given the gene expression vectors of the learned pseudo-perturbations, multiple cells from each studied stage can have the same gene expression value. We call these cells *redundant cells*. In fact, for the set of cells, ${{\mathcal{R}}}^{A}$, composed of pseudo-perturbation cells and redundant cells for stage *A*, only one binarized gene expression profile should be considered for the set of *k* genes selected in the pseudo-perturbation learning step. We therefore generate two sets of cells, ${{\mathcal{R}}}^{A}$ and ${{\mathcal{R}}}^{B}$, that include the pseudo-perturbation cells and the redundant cells for each developmental stage.

We use them to select gene expression values for the *readout nodes* that maximize the difference between both stages. For this, we iterate across all pairs of cells in ${{\mathcal{R}}}^{A}\times {{\mathcal{R}}}^{B}$ and calculate the difference between the readout gene values, retaining the pair with the maximal difference.

Additionally, we calculate the *representativity score* for each studied stage to provide objective information about the representativity of the identified pseudo-perturbations. Considering *n*^*A*^ as the number of cells in stage *A*, the representativity score *S*^*A*^ is the proportion of cells in ${{\mathcal{R}}}^{A}$ defined as follows:

3 $${S}^{A}=\frac{| {{\mathcal{R}}}^{A}| }{{n}^{A}}.$$

### BN inference

Once the PKN reconstructed and stage-specific experimental designs are constructed, we use the Caspo tool^11^ to infer stage-specific families of BNs. The PKN provides the set of possible gene interactions that can occur in the system, while the experimental design, consisting of pseudo-perturbations and pseudo-observations, captures the gene expression observed in the system at specific layers of the PKN (input, intermediate and readouts). Using logical rules and constraints in ASP, Caspo searches to infer the minimal BN that models both the PKN interactions and the gene expression. The BN inference process is based on a fitting distance called mean squared error (MSE). Through the inferred BN, the expression of pseudo-observations is predicted and compared to the real values provided by the experimental design. The distance between these two values is the MSE. To infer families of BNs for a specific stage, the user can modulate the search space by providing a tolerance regarding the fitting distance (*fitness*_*tolerance*) and a second tolerance in terms of the size of the BNs (*size*_*tolerance*), allowing the inference of suboptimal BNs with a larger number of interactions than the optimal one. Furthermore, a length parameter (*length*) is provided to fix the number of incoming edges to each logical “and” gate. For further information about Caspo, please refer to Videla et al.^11^.

For our case study analyzing TE and mature TE, we apply the arctangent normalization on to obtain the solution 1 pseudo-perturbation. We set the following parameter values for the BN inference: *fitness*_*tolerance* = 0.0001, *size*_*tolerance* = 0 and *length* = 2.

### Cell classifier

Recall that the BN inference using Caspo is based on predicting readout expression through the BN. The metric used for this prediction is the mean square error (MSE), which represents the distance between pseudo-observation prediction values and observed expression values. We use this MSE metric to implement a *cell classifier*, aiming to sort cells into a developmental stage. Our classifier follows this procedure:

- **Distance computation:** Given a testing cell *c*, two stages *A* and *B* with their respective learned BNs: *BN*^*A*^, *BN*^*B*^, the algorithm computes the weighted MSE of the readout predictions, given the input-intermediate gene expression values of *c* using both BNs. This yields two distances representing the discrepancy between pseudo-observation predictions and observations for *c*: *MSE*^*A*^(*c*) and *MSE*^*B*^(*c*).
- **Classification:** The algorithm assigns to *c*, the stage (*A* or *B*) with the smallest error distance. Cells where *MSE*^*A*^(*c*) = *MSE*^*B*^(*c*) are not classified.

Given a set of testing cells we want to classify, we calculate the following metrics:

- *Class-specific accuracy:* provides the percentage of accuracy (*i.e*., correct classification) for a specific stage.
- *Global accuracy:* provides the average accuracy between the two studied stages.
- *Balanced accuracy (BAC):* provides a balanced accuracy considering the number of cells within each stage.

We evaluate the performance of the cell classifier by applying a threshold on the Mean Squared Error (MSE) to exclude ambiguous cases. The goal is to filter out cells where classification confidence is questionable based on their MSE values. To achieve this, we define an exclusion interval [*MSE* − *ε*, *MSE* + *ε*] around the MSE threshold, where *ε* is a tunable parameter. Any cell with an MSE within this interval is considered ambiguous and is therefore not classified. This allows us to assess the impact of different levels of uncertainty filtering on classifier performance. We test different values of *ε* ({0, 0.01, 0.05, 0.1}) and analyze their effect on key metrics, including overall accuracy, BAC, and the proportion of cells that remain unclassified due to falling within the exclusion interval. The results are presented in Supplementary Fig. 3.

## Supplementary information

Supplementary Information

## Data Availability

The datasets analyzed and the produced results during the current study are available in the zenodo repository: 10.5281/zenodo.14198406.

## Code availability

The SCIBORG method presented in this study is an open-source Python package with a command line interface, publicly available at the following link: https://github.com/mathieubolteau/SCIBORG.
