# Supplementary material for: Deep exploration of logical models of cell differentiation in human preimplantation embryos
Source: NPJ Syst Biol Appl. 2025 May 27;11:57. doi: 10.1038/s41540-025-00537-7 (PMC12117111; doi:10.1038/s41540-025-00537-7)
Supplement: Supplementary file 1 — Supplementary Information [file 41540_2025_537_MOESM1_ESM.pdf]

Deep exploration of logical models of cell differentiation in human preimplantation embryos

Supplementary information

Contents

1 Supplementary Fig. 1. Learned BNs for $k = 15$. 1

2 Supplementary Fig. 2. Learned BNs of Solution 2. 2

3 Supplementary Fig. 3. Cell classifier with threshold of non-considered MSE. 3

4 Supplementary Fig. 4. Gene expression profiles. 4

5 Supplementary Fig. 5. Expression profiles in comparison of NR2F2. 5

6 Supplementary Fig. 6. Reconstructed PKN. 6

7 Supplementary Table 1. Pseudo-perturbation identification program comparison. 8

8 Supplementary Note 1. State-of-the-art modeling method comparison. 9

9 Supplementary Note 2. Expansion of testing scenarios. 11

10 Supplementary Note 3. Queries on Pathway Commons using pyBRAvo. 13

11 Supplementary Note 4. Transcription factor gene list used for PKN reconstruction. 14

12 Supplementary Note 5. Line-by-line explanation of the pseudo-perturbation identification program. 15

13 Supplementary Note 6. Logical rules that make up the learned BNs. 18

1 Supplementary Fig. 1. Learned BNs for $k = 15$.

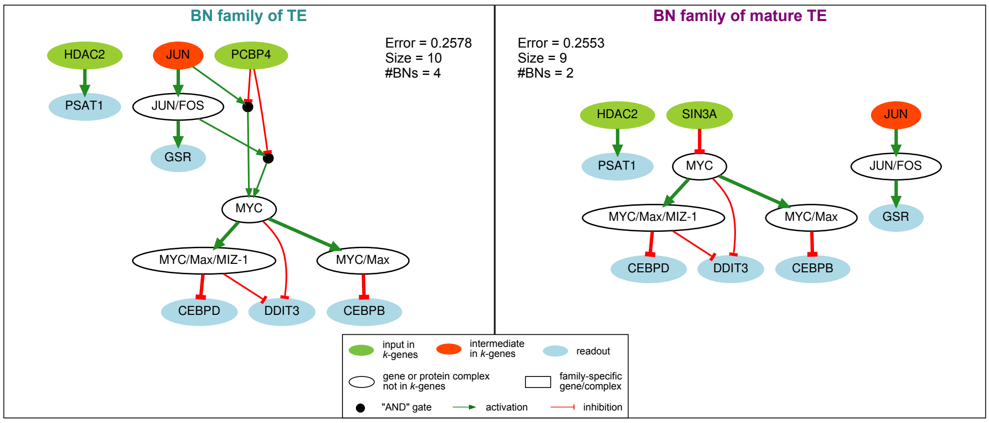

Supplementary Fig. 1 Learned BNs for $k = 15$. Families of learned BNs for TE and mature TE stages for $k = 15$. Each network represents the union of (sub-)optimal BNs learned from the reduced PKN and the experimental design. The colored nodes represent genes associated with experimental designs, including input and intermediates involved in pseudo-perturbations, and readout genes involved in pseudo-observations. The width of the arc represents the frequency of occurrence of this interaction in the BNs. We set the following parameter values for the BN learning: $fitness\_tolerance = 0.0001$, $size\_tolerance = 0$ and $length = 2$.

2 Supplementary Fig. 2. Learned BNs of Solution 2.

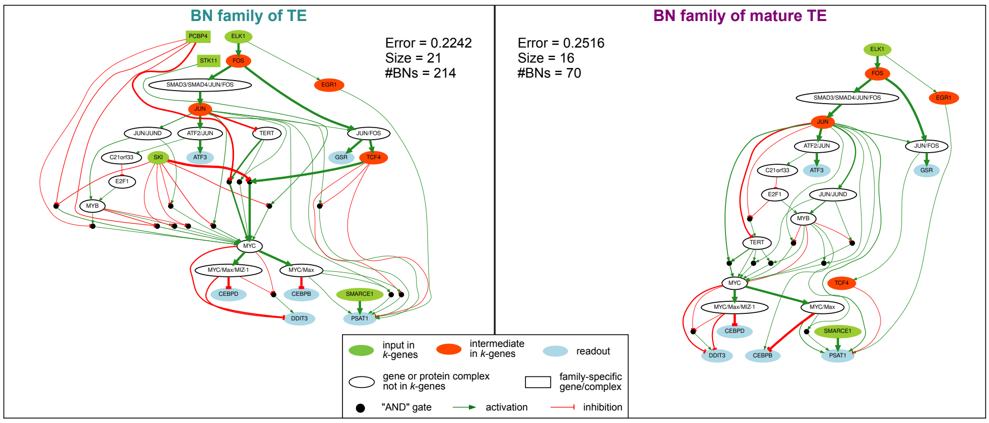

Supplementary Fig. 2 Learned BNs of Solution 2. Families of learned BNs for TE and mature TE stages. Each network represents the union of (sub-)optimal BNs learned from the reduced PKN and the experimental design. The colored nodes represent genes associated with experimental designs, including input and intermediates involved in pseudo-perturbations, and readout genes involved in pseudo-observations. The width of the arc represents the frequency of occurrence of this interaction in the BNs. We set the following parameter values for the BN learning: $fitness\_tolerance = 0.0001$, $size\_tolerance = 0$ and $length = 2$.

3 Supplementary Fig. 3. Cell classifier with threshold of non-considered MSE.

a

Solution 1

Pseudo-perturbation cells (training set)

Epsilon	Accuracy	BAC	TE accuracy	Mature TE accuracy	#cell not predicted Total	TE	Mature TE
0	72	72	64	81	0	0	0
0.01	79	78	67	89	24	13	11
0.05	77	77	70	85	66	29	37
0.1	79	79	74	84	80	34	46

Redundant cells (testing set 1)

Epsilon	Accuracy	BAC	TE accuracy	Mature TE accuracy	#cell not predicted Total	TE	Mature TE
0	66	67	52	82	0	0	0
0.01	68	69	52	86	31	16	15
0.05	69	70	57	83	99	45	54
0.1	71	71	59	83	122	61	61

Other cells (testing set 2)

Epsilon	Accuracy	BAC	TE accuracy	Mature TE accuracy	#cell not predicted Total	TE	Mature TE
0	68	68	62	73	0	0	0
0.01	69	70	63	77	10	2	8
0.05	71	71	73	68	56	18	38
0.1	72	70	77	63	78	29	49

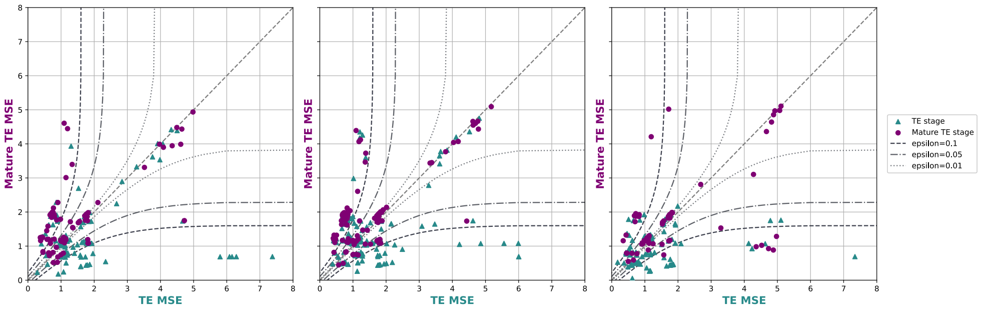

b

Solution 2

Pseudo-perturbation cells (training set)

Epsilon	Accuracy	BAC	TE accuracy	Mature TE accuracy	#cell not predicted Total	TE	Mature TE
0	78	78	62	94	88	44	44
0.01	78	78	62	94	89	44	45
0.05	80	79	64	94	94	49	45
0.1	82	82	73	92	114	56	58

Redundant cells (testing set 1)

Epsilon	Accuracy	BAC	TE accuracy	Mature TE accuracy	#cell not predicted Total	TE	Mature TE
0	68	73	50	96	83	26	57
0.01	69	74	50	98	86	28	58
0.05	70	74	52	97	100	37	63
0.1	81	82	68	97	139	68	71

Other cells (testing set 2)

Epsilon	Accuracy	BAC	TE accuracy	Mature TE accuracy	#cell not predicted Total	TE	Mature TE
0	67	68	53	84	74	32	42
0.01	67	69	53	85	78	34	44
0.05	71	73	56	90	84	37	47
0.1	79	80	68	91	105	53	52

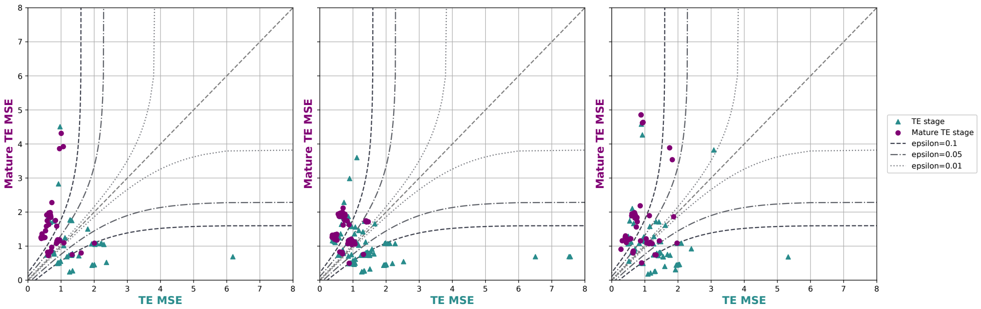

Supplementary Fig. 3 Cell classifier with threshold of non-considered MSE. We evaluate different threshold values (epsilon = 0, 0.01, 0.05, 0.1) to define an exclusion interval. For each threshold, we compute classification accuracy, balanced accuracy (BAC), and the number of cells excluded from classification due to their MSE values falling within the threshold range. Negatively log-transformed MSE values. **a** Solution 1. **b** Solution 2.

4 Supplementary Fig. 4. Gene expression profiles.

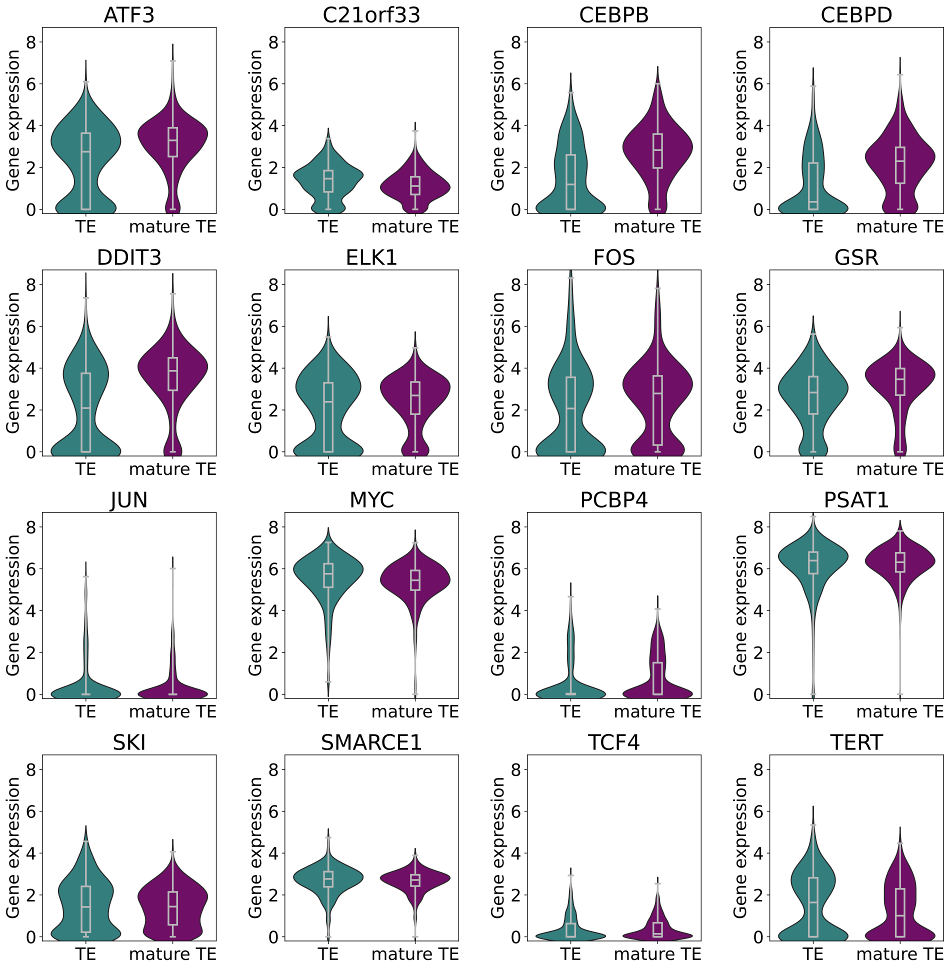

Supplementary Fig. 4 Gene expression profiles. The expression profiles of all genes included in the inferred BNs (see Fig. 4). Log-transformed gene expressions (see Methods).

5 Supplementary Fig. 5. Expression profiles in comparison of NR2F2.

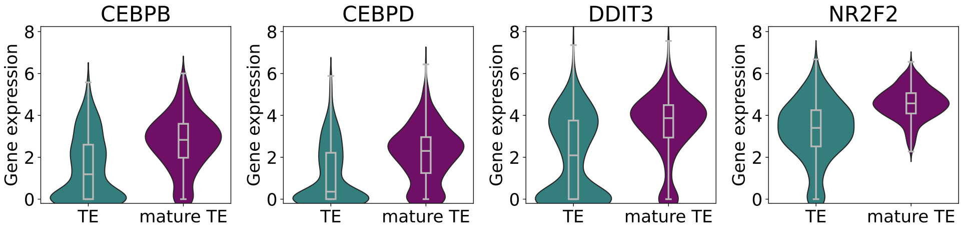

Supplementary Fig. 5 Expression profiles in comparison of NR2F2. Log-transformed gene expressions (see Methods).

6 Supplementary Fig. 6. Reconstructed PKN.

See next page.

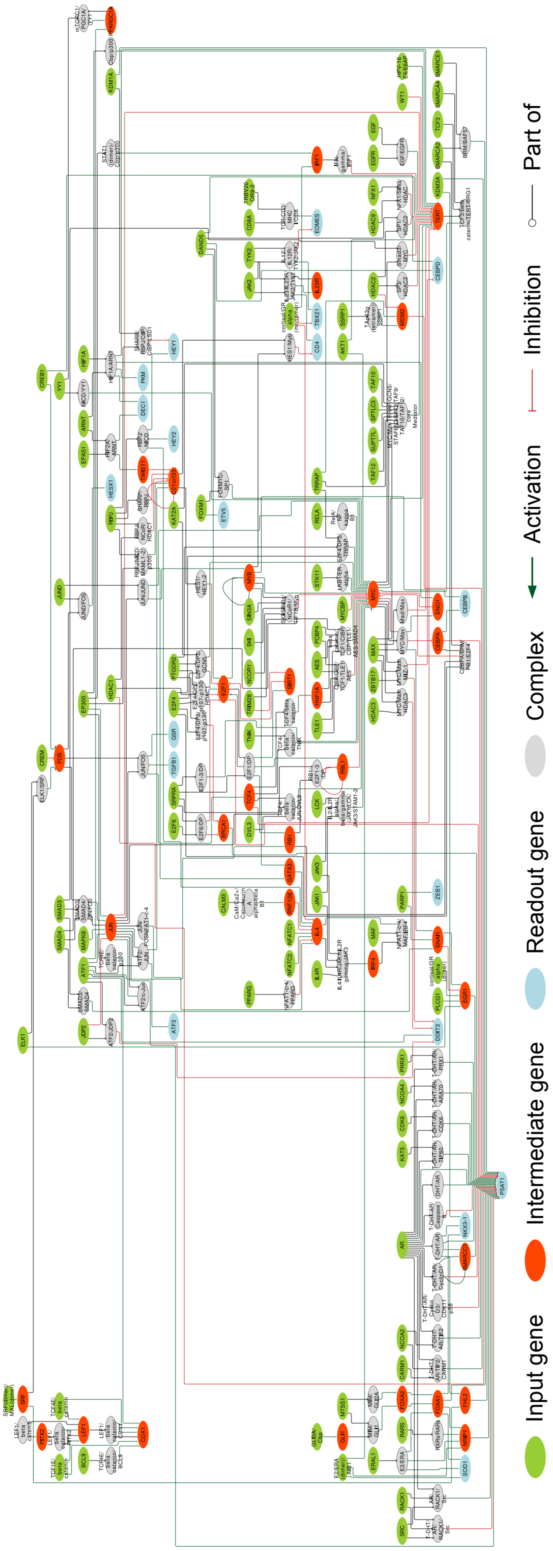

Supplementary Fig. 6 Reconstructed PKN. The PKN was reconstructed via the query of Pathway Commons using pyBRAvo (see Methods). We use a list of 438 transcription factors as input (Supplementary Note 4. Transcription factor gene list used for PKN reconstruction). The reconstructed PKN comprises 225 nodes and 369 edges. Among the nodes, we identify 85 input genes, 36 intermediate genes, 19 readout genes, and 85 protein-complexes.

7 Supplementary Table 1. Pseudo-perturbation identification program comparison.

Supplementary Table 1 Pseudo-perturbation identification program comparison.

Dataset	k	$l^{\dagger}$	Chebouba et al version [1]		Bolteau et al version [2]		SCIBORG version	
			Execution time	Pseudo-perturbations	Execution time	Pseudo-perturbations	Execution time	Pseudo-perturbations
A	3	2	0.008s	3	0.008s	3	0.009s	3
B	3	2	0.048s	1	0.223s	4	0.060s	3
C	3	2	1.420s	1	15 min*	7	1.168s	6
	10	6	1.424s	1	15 min*	11	15min*	13
D	10	6	15 min*	10	15 min*	27	15min*	35
SC	10	6	5h 2 min	3	65h*	20	**7h***	**92**
P	10	NA	50h*	23	50h*	25	50h*	30

We report the number of pseudo-perturbations identified across the different datasets within a certain execution time, for specific values of k and l (see Methods). For the Chebouba et al. version, a post-processing step was performed to remove redundant pseudo-perturbations, which is not part of this program. For the phosphoproteomic dataset (P), no inputs are used (NA), and the ASP constraints related to inputs are removed. † Considered parameter only for SCIBORG version. * Execution time corresponds to the fixed timeout. Executions were led on a computer cluster.

For an equal runtime on dataset D, the SCIBORG version identifies 3.5 times more pseudo-perturbations than the Chebouba et al. version (35 vs. 10) and 1.3 times more than the Bolteau et al. version (35 vs. 27). On the larger dataset SC, SCIBORG detects 4.6 times more pseudo-perturbations than Bolteau et al. (92 vs. 20) while requiring 9 times less computation time (65h vs. 7h). These results demonstrate that the SCIBORG version is an order of magnitude faster.

8 Supplementary Note 1. State-of-the-art modeling method comparison.

In this Supplementary Note, we compare the modeling methods presented in the article (Supplementary Table 2).

Supplementary Table 2 Comparison of modeling methods.

Method	Input content	Input size	Prior knowledge	Cell heterogeneity	Developmental evolution	Exhaustive enumeration	Global search
SCNS [3]	• Single-cell transcriptomics	• ≈ 40 genes<br>• ≈ 4,000 cells	• TFs implicated in the system	✓	✓	✓	✗
RE:IN [4]	• Abstract BN<br>• Perturbation observations	• ≈ 20 genes<br>• ≈ 30 perturbations	• Potential gene interactions<br>• TFs implicated in the system	✗	✗	✗	✓
WASABI [5]	• Temporal scRNA-seq<br>• Bulk Proteomics	• ≈ 100 genes<br>• ≈ 96 cells	• None	✗	✓	✓	✗
BoNesis [6]	• scRNA-seq<br>• PKN<br>• Dynamical properties	• ≈ 1,000 genes<br>• ≈ 600 cells	• Potential gene interactions<br>• Dynamical constraints	✗	✓	✗	✓
SCIBORG (this study)	• scRNA-seq<br>• PKN	• ≈ 150 genes<br>• ≈ 700 cells	• Potential gene interactions	✓	✓	✓	✓

These methods infer Boolean (SCNS, RE:IN, BoNesis and SCIBORG) or ODE-based (WASABI) models that satisfy a set of given constraints or equations; providing mechanistic and predictive models. They all tackle combinatorial search problems, and their solutions are inferred by using logic or numerical solvers. We have focused on comparing four type of characteristics that we consider essential for providing quality BNs and that approach single-cell data specificity:

- *Cell heterogeneity*, which is the ability to include diverse gene expression states, in a subset of identified genes, and in all cells.
- *Developmental evolution*, which is the ability to propose an understanding of the transition between at least two developmental stages.
- *Exhaustive-robust enumeration*, when methods tackle the inference of logic or numerical functions by exploring the entire set of solutions.
- *Global search*, when methods addresss the inference of (logical) regulatory functions with a global search approach; in contrast to local search methods where regulatory functions will be derived individually for each node.

We discuss now the ability of each method to approach or not these characteristics.

SCNS focuses on a small evolutionary window of the system studied (40 TFs). Their approach will infer the Boolean update functions for each gene individually from the state transition graph derived from single-cell data analysis. Their model approaches the transition between two developmental stages. The cut of the search space explosion is done by fixing parameters to the number of literals within each update Boolean function; they do not start from a fully connected model. Resultant and optimal Boolean functions are exhaustively explored independently for each gene, and those above a specific threshold are kept. The resultant Boolean network from this approach is highly interconnected and little structured; this may be a result of the local search of Boolean functions.

RE:IN is a method that derives Boolean networks from perturbation data. It has been used to study mouse embryonic development through a series of multiple knock-outs. The models they propose represent embryos in a particular developmental stage but single-cell data is not used at the level of model inference. This method does not model nor approach the evolution between two or more developmental stages. Tackling, as all other cited methods, a combinatorial search problem, only a part of the solutions is considered.

WASABI infers ODE-based models from single-cell time-series data, incorporating a post-transcriptional step into the model, which adds an interesting layer of complexity. Using numerical methods applied independently to each node, the approach prunes an initially fully connected graph by assigning directions, signs, and kinetic constants that align with experimental observations. This results in models that explain transitions between developmental stages by using numerical approximations of the parameter space to define the ODEs. To handle complexity, computations are parallelized, allowing independent processing of graph nodes. As demonstrated in [5], the resulting models exhibit low inter-connectivity and a lack of multiple regulators.

BoNesis is designed to infer dynamical Boolean networks that model transitions across various developmental stages. By incorporating prior knowledge of gene regulation—similar to approaches like RE:IN and SCIBORG—the resulting Boolean networks exhibit a clear topological structure. However, these methods require collapsing the heterogeneity of gene expression among cells at the same stage to produce an average expression profile. Since they address a satisfiability problem, they generate billions of solutions but do not exhaustively explore the entire solution space.

Recall that our problem was to understand and model mechanisms involved in the human preimplantation embryo development. When putting the aforecited methods in perspective with our problem we notice:

- The SCNS framework meets mostly all of our expectations. It addresses cellular heterogeneity, two developmental stages evolution, and tackle exhaustivity in the solution space. However, its local search of Boolean function updates may infer models lacking of structure. In our context we were interested to discover Boolean models of gene regulatory networks, known to have properties such as hierarchical organization and sparsity [7], and this is not met by SCNS.
- The RE:IN method's input requirements are restrictive, relying on constraints from biological experiments involving activation or inhibition. In the context of human embryos, these system perturbations are infeasible due to various factors, including legal ones.
- WASABI is not feasible for us since we do not count in our biological system with time-series experimental data.
- The BoNesis method's approach of analyzing subsets of cells might exclude some cells of the same type, potentially omitting valuable gene expression data. Additionally, calculating the observation sign from an average of cells might dilute unique gene expression patterns, potentially missing interesting behaviors.

In conclusion, we introduced SCIBORG, a novel framework designed to infer Boolean network models that distinguish between different developmental stages in human embryonic development using single-cell data. Like WASABI, SCIBORG can be extended to incorporate post-transcriptional events by querying the Pathway Commons database. However, unlike the referenced methods, our approach infers BNs for two distinct stages and establishes frameworks that leverage these BNs as cell classifiers—enhancing both the orientation and discovery of new networks.

Overall, SCIBORG constructs logical models of regulatory mechanisms across two developmental stages, providing deeper insights into cell transitions and lineage commitment. The framework supports exhaustive model enumeration and optimality searches, while addressing cell heterogeneity and redundancy. The inferred models are assessed using a precision metric, thereby strengthening confidence in the robustness of the results.

9 Supplementary Note 2. Expansion of testing scenarios.

In this section, we present another analysis we performed on different developmental stages. We compared B1-B2 (blastocyst 1-2) with early TE to complete the overview of the TE specification branch. We computed the number of pseudo-perturbations found in 7 days and a BN family for each stage. The number of k-selected genes was set to 10. We note that the stages explored in this part do not comprise as many cells as the stages mentioned in the main article (TE and mature TE). There were 171 and 121 cells for B1-B2 and early TE respectively whereas TE and mature TE comprised 348 and 332 cells, respectively. A lower number of cells means less pseudo-perturbations possible. As we can see in Supplementary Fig. 8, 53 pseudo-perturbations are identified for B1-B2 and early TE, after an initial exponential increase phase followed by a plateau. The results converge which shows they are close to optimality.

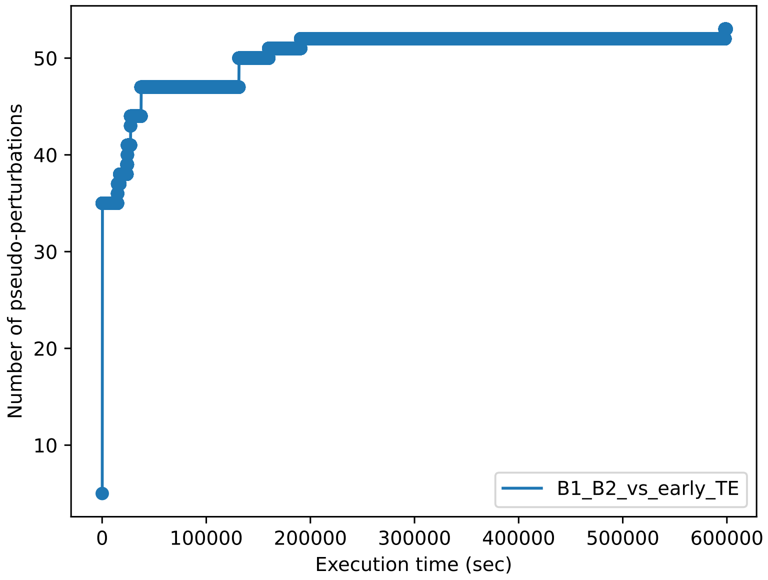

Supplementary Fig. 7 Convergence of the identified pseudo-perturbations over time in B1-B2 vs early TE scenario. The results were obtained using a computer cluster. The computation was stopped after 7 days. 3 equivalent solutions were found. *Number of cells* : 171 (B1-B2), 121 (early TE).

Learned BNs for all pairs of studied developmental stages are visible in Supplementary Fig. 8. B1-B2 and early TE have a similar amount of logical gates (13 and 14 respectively). In terms of complexity, B1-B2 seems more complex to model than early TE, with the information from our 53 pseudo-perturbations, as shown by its higher error (0.2791 > 0.2343) (Supp. Fig. 8c). Focusing on B1-B2 and early TE families (Supp. Fig. 8c), the same number of inputs and intermediates is observed, 5 and 6 respectively. We note that the intermediates are the same but not the inputs. Only *MAPK8*, *SNAI1* and *MTSS1* are common inputs. The readouts are also similar with the B1-B2 family having one more readout, *DDIT3*. We can also see similar structures between the two families. For example, 2 of the 3 branches of the cascade starting with *MAPK8* are common to both stages: activations of *ATF3* and *GSR*. The *MTSS1* cascade to activate *SOD1* is also shared by both families. The main difference is the activation/inhibition of *PSAT1*. The mechanisms are more direct in early TE models while more "AND" gates involving *EGR1* are present in B1-B2 BNs.

This analysis provides new trails to explore and validate experimentally and shows the adaptability of our method. SCIBORG can be applied to other developmental stages but also to other biological contexts and processes.

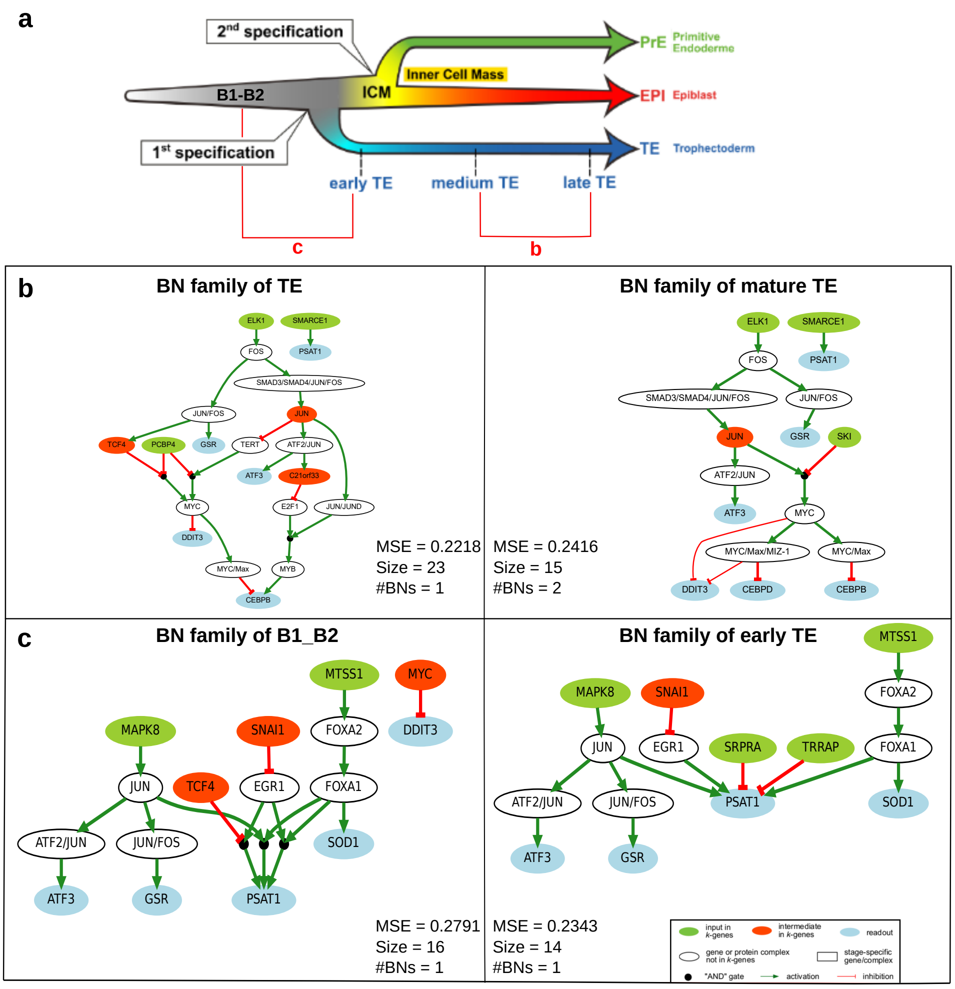

Supplementary Fig. 8 BNs inferred for different scenarios. **a** Overview of human pre-implantation development stages. The three cell layers EPI, PrE and TE drift from two distinct specifications. The TE branch is made of three stages : early TE, medium TE (also called TE) and mature TE. The letters in red reference the sub-figures showing the corresponding learned BNs. Figure adapted from [8]. **b** Families of learned BNs for TE and mature TE stages, also in Fig. 4. **c** Families of learned BNs for B1-B2 and early TE stages.

In all scenarios, each network represents the union of (sub-)optimal BNs learned from the reduced PKN and the experimental design. Genes associated with experimental designs such as input, intermediates found in pseudo-perturbations and readouts from pseudo-observations are represented by colored nodes. The width of the arc represents the frequency of occurrence of this interaction in the BNs. We set the following parameter values for the BN learning: $fitness\_tolerance = 0.0001$, $size\_tolerance = 0$ and $length = 2$.

10 Supplementary Note 3. Queries on Pathway Commons using pyBRAvo.

To make queries on Pathway Commons (PC), pyBRAvo requires a SPARQL endpoint, a system to execute the query and return the result. At the moment of the study, the endpoint hosted by PC was not available. Consequently, we create a local endpoint to execute the query on PC data.

1. Download PC data We first download the whole PC data from the PC website using the link: https://download.baderlab.org/PathwayCommons/PC2/. We use the PC version 12, the last version available during our study. After choosing the wanted version, we download the file "pc-biopax.owl.gz" containing all the PC data.

2. Parameterize a database management system To host PC data and allow queries, a database management system is required. We use GraphDB (https://graphdb.ontotext.com) as the management system. After installation, we create a new repository and then import the previously downloaded file containing PC data. Once imported and the repository is running, the URL of the SPARQL endpoint can be copied and used by pyBRAvo.

3. Execute query The URL provided by GraphDB should be used in the "endpoint" parameter of the configuration file of SCIBORG to parametrize pyBRAvo package. The PKN can be then reconstructed through SCIBORG using the command `--pkn-reconstruction` (see documentation of the software).

11 Supplementary Note 4. Transcription factor gene list used for PKN reconstruction.

Below, we present the list of the 438 transcription factor genes involved in human embryonic development given as input for PKN reconstruction.

ACO1	EN1	ING3	NOBOX	SNAI3	ZFP64	ZNF557
AKR1A1	ERG	IRF3	NR1H4	SND1	ZFP90	ZNF559
ANXA1	ESRRG	IRF4	NR2E1	SOD1	ZHX2	ZNF561
ANXA11	ETFB	IRF6	NR2F2	SOX1	ZHX3	ZNF569
ARG2	ETS2	IRF8	NR3C1	SOX11	ZIC3	ZNF574
ARID5B	ETV1	IRX2	NR3C2	SOX15	ZIK1	ZNF578
ASH2L	ETV4	IRX3	NR4A3	SOX17	ZIM2	ZNF584
ATF2	ETV5	IRX4	NR5A2	SOX2	ZIM3	ZNF586
BACH2	EXO5	IRX5	NR6A1	SOX30	ZKSCAN4	ZNF595
BARHL2	EZR	ISL2	NRF1	SOX4	ZKSCAN5	ZNF596
BARX1	FEZF2	JAZF1	OLIG1	SOX5	ZNF10	ZNF597
BARX2	FHL2	JDP2	OSR1	SOX9	ZNF117	ZNF599
BATF	FIGLA	JRKL	OSR2	SP110	ZNF132	ZNF606
BCL11A	FLI1	KDM4A	OTX1	SP6	ZNF134	ZNF610
BCL3	FOSB	KDM4D	OTX2	SPIC	ZNF136	ZNF616
BHLHE40	FOSL1	KDM4E	OVOL1	SSX3	ZNF140	ZNF630
CARF	FOXA2	KLF11	OVOL2	STAT3	ZNF146	ZNF654
CBFA2T2	FOXD2	KLF12	P4HB	STAT5A	ZNF155	ZNF668
CCDC25	FOXN2	KLF17	PARP1	SUCLG1	ZNF157	ZNF669
CDX1	FOXO3	KLF18	PAX9	TAF7	ZNF16	ZNF674
CDX2	FOXP1	KLF2	PBX3	TAGLN2	ZNF165	ZNF675
CEBPA	FOXQ1	KLF3	PIR	TBPL2	ZNF17	ZNF677
CEBPB	GATA2	KLF4	PITX2	TBX2	ZNF174	ZNF679
CEBPD	GATA3	KLF6	PKM	TBX3	ZNF18	ZNF684
CELF5	GATA4	KLF7	PKNOX2	TBX5	ZNF182	ZNF689
CERS2	GBX1	KLF9	PLAG1	TCF24	ZNF184	ZNF69
CERS3	GBX2	KLRG1	PLAGL1	TCF7L1	ZNF19	ZNF697
CERS6	GCM1	LARP1	POLD2	TCF7L2	ZNF200	ZNF701
CLOCK	GIT2	LEF1	POU5F1B	TEAD1	ZNF211	ZNF702P
CPrEB1	GOT1	LHX2	PPARG	TEAD3	ZNF214	ZNF705A
CREB5	GPD1	LHX5	PPARGC1A	TFAP2A	ZNF215	ZNF705CP
CREBL2	GRHL1	LHX8	PRDM1	TFAP2B	ZNF226	ZNF705D
CTBP1	GRHL2	LRRFIP1	PRDM10	TFAP2D	ZNF23	ZNF705G
CTCFL	GRHL3	LSM6	PRDM11	TFCP2L1	ZNF230	ZNF706
CTNNB1	GRHPR	LUZP2	PRDM14	TFEB	ZNF25	ZNF708
CUX2	GTF2A1L	MAF	PRDM16	TGIF2LX	ZNF256	ZNF714
CYB5R1	GTF3A	MCTP2	PRDX5	THAP1	ZNF266	ZNF716
DAB2	H2AFY	MECOM	PRKAA1	THRB	ZNF280A	ZNF727
DBP	HAND1	MIXL1	PRKAA2	TIGD2	ZNF284	ZNF735
DDIT3	HCFC2	MLXIPL	PSMC2	TOPORS	ZNF304	ZNF736
DDX4	HES1	MSI2	PSMD12	TP63	ZNF329	ZNF766
DDX43	HESX1	MSRA	RAB14	TPI1	ZNF331	ZNF79
DLX1	HEY1	MSRB3	RAB18	TPPP	ZNF341	ZNF814
DLX2	HEY2	MTHFD1	RARB	TRIB2	ZNF343	ZNF829
DLX3	HHAT	MYCL	RAX2	TRIB3	ZNF350	ZNF830
DLX4	HHEX	MYLK	RBBP9	TRIP10	ZNF354A	ZNF831
DLX5	HIF1A	NANOG	RELB	TULP1	ZNF362	ZNF844
DMRTB1	HIRIP3	NANOGP8	RFX4	UBE2V1	ZNF385A	ZNF845
DMRTC2	HIST1H2BN	NCALD	RFXANK	UGP2	ZNF394	ZNF878
DNMT1	HIST2H2BE	NEUROG2	RLF	VENTX	ZNF408	ZNF880
DPRX	HKR1	NFATC1	RORB	YWHAZ	ZNF416	ZNF891
DUXA	HLF	NFE2	RUNX1	YY2	ZNF438	ZNF92
E2F8	HNF1A	NFIX	RUNX2	ZBTB11	ZNF439	ZRSR2
EBF1	HNF4A	NFKB1	RUVBL1	ZBTB16	ZNF440	ZSCAN10
EBF2	HOXA4	NFKB2	RXRA	ZBTB49	ZNF479	ZSCAN18
EBF3	HOXA7	NFYA	SALL2	ZBTB7B	ZNF490	ZSCAN32
ECSIT	HOXA9	NKX2-5	SATB1	ZCCHC14	ZNF506	ZSCAN4
EGR1	HOXB13	NKX3-1	SETBP1	ZEB1	ZNF528	ZSCAN5A
EGR2	HOXB6	NKX3-2	SHOX2	ZFHX3	ZNF530	ZSCAN5B
EIF5A2	HOXC10	NKX6-1	SIN3A	ZFP3	ZNF534	ZSCAN5C
ELF3	HOXD8	NKX6-2	SMAD5	ZFP37	ZNF541	
ELK3	HTATIP2	NMI	SMAD6	ZFP42	ZNF549	
EMX1	HUNK	NNT	SNAI1	ZFP62	ZNF555	

12 Supplementary Note 5. Line-by-line explanation of the pseudo-perturbation identification program.

We provide a step-by-step description of ASP program of pseudo-perturbation identification presented below.

```
1  {selinput(G) : pert(C,G,S,CL), not intermediate(G)} = l.
2  {selinter(G) : intermediate(G)} = k-l.
3  selpert(E,V,S,C) :- selinput(V), pert(E,V,S,C).
4  selpert(E,V,S,C) :- selinter(V), pert(E,V,S,C).
5  equal(I,J,G) :- selpert(I,G,S1,C1), selpert(J,G,S2,C2), I!=J, S1=S2.
6  pot_match(I,J) :- k = {equal(I,J,_)}, selpert(I,_,_,C1),
       selpert(J,_,_,C2), C1<C2, I!=J.
7  0{match(I,J)}1 :- pot_match(I,J).
8  nbInputOnes(C,N) :- N = {pert(C,G,1,_) : selinput(G), input(G)},
       match(C,_).
9  :- match(C,_), nbInputOnes(C,N), N < 1.
10 :- match(I,J1), match(I,J2), J1!=J2.
11 :- match(I1,J), match(I2,J), I1!=I2.
12 :- pot_match(I1,J), pot_match(I2,J), match(I2,J), I1<I2.
13 :- pot_match(I,J1), pot_match(I,J2), match(I,J2), J1<J2.
14 1{selinput(G) : ancestor(G,I), input(G)} :- selinter(I).
15 #maximize{1, I : match(I,_)}.
```

Recall that the program objective is to maximize the count of pseudo-perturbation matches between different class cells, respecting rules and constraints outlined in the Methods section of the paper. Here, a match refers to identical Boolean expression values for a set of genes between two cells from different classes. We refer to these Boolean values as a pseudo-perturbation.

First, experimental data is formulated using `pert/4` predicates, giving the expression of a gene G at value S in cell C, linked to class CL. The program starts by selecting a set of l input genes from all genes that are not intermediate ones using the line 1. Line 2 selects a set of $k - l$ intermediates genes. Together, these rules ensure respect for the *Constraint 1*.

Once genes are selected, we filter experimental data using the `selpert/4` predicate. For each selected input (resp. intermediate), a predicate is defined in line 3 (resp. 4).

Then, line 5 associates with the `equal/3` predicate a pair of cells I and J having the same expression for the gene G (`S1==S2`). Line 6 defines a potential match (`pot_match/2` predicate) between two distinct cells I and J (`I!=J`) if there exist k genes having the same expression for these two cells (`equal/3` predicate). These cells should be part of a different class (`C1<C2`). This rule means that cells involved in a `pot_match/2` predicate could form a match because they have the same expression for the k selected genes. A potential match could lead to a match or not. This is the purpose of the line 7 where, given a `pot_match/2` predicate, a `match/2` can be inferred or not, via the choice rule `0{...}1`.

Afterward, the program handles data sparsity by defining `nbInputOnes/2`, counting for a cell C involved in a match, the number of expressed input genes. With the constraint line 9, the program forbids a match comprising a cell having less than 1 expressed input. Here, the program ensures that each pseudo-perturbation has at least one expressed input gene (referred to as *Constraint 3*).

Lines 10 to 13 answer to *Constraint 2*, by preventing potential redundancies in a cell class. The objective is to forbid the selection of cells within the same class having the same k-genes expression. For that, we implement two constraints (lines 10 and 11) that forbid solutions where the same cell is present in two `match/2` predicates, in the first class (resp. second class) with line 10 (resp. 11).

To better illustrate this, let us consider a toy example comprising 3 cells in class A and 3 cells in class B following the "V-patterns" presented in Supplementary Fig. 3a. The cell $a1$ may match with $b1$ and $b2$, and cell $b3$ may match with $a2$ and $a3$, representing in total 4 potential matches. Given the line 7, a potential match could infer a match for the considered pair of cells. Thus, $2^4 + 1$ possible solutions could be deduced. The additional solution corresponds to the empty set, which signifies inferring zero matches. An example of an induced solution could be the configuration where all 4 potential matches are inferred as matches (Supplementary Fig. 3a). However, this configuration contains redundancies that must be avoided. This is achieved with the constraints lines 10 and 11 forbidding V-patterns in all solutions. Therefore, the program generates 9 possible solutions having 0, 1 or 2 matches.

The two constraints in lines 10 and 11 prohibit redundancies of V-patterns; however, one configuration does not comply with what is required. This configuration is created when multiple cells within a class match with multiple cells within the other class. We illustrate in Supplementary Fig. 3b the simpler case where 2 cells of a class have the same k-gene expression of 2 cells of another class. In this case, we have 4 potential matches, but we want to yield only one of them because they are all equivalent. After applying the 10 and 11 rules, we obtain 2 patterns comprising 2 matches: the "parallel-pattern" and the "cross-pattern". To overcome this, we introduce two constraints (lines 12 and 13). These two constraints are complementary. The first, in line 12, prohibits a solution where 2 cells ($I1$ and $I2$) potentially match with a cell J and a match between $I2$ and J is considered. The idea behind this is to only keep the match between $I1$ and J, by filtering cross- and parallel-patterns of $I2$. The second, in line 12, is similar to the first one, filtering cross- and parallel-patterns of the opposite symmetry. These constraints reinforce to keep on this type of configuration **b**, only 1 match: `match(a1,b1)`.

Two remarks are necessary. First, if the constraints 12 and 13 are applied to configuration A (V-patterns), only 1 solution will be admissible (out of the 9 possible after 10 and 11): `{match(a1,b1), match(a2,b3)}`. Second, it is important to note that our program is very constraining, drastically limiting the number of equivalent solutions. While other possible matches between cells exist, they are redundant. We explore these redundancies afterward using a Python program in a later step called "readout difference maximization" (see the Methods section of the paper for more details).

Comparing this ASP program version with the one presented previously in Bolteau et al. [2] for the redundancies handling, the last version introduces 2 new predicates that are costly for the solver, especially the `countdiff/3` one which requires a costly choice rule. Note that the grounder needs to enumerate all possible sets that respect the rules in a choice rule, which is highly resource-consuming in terms of execution time and storage. Current version uses only constraints that are not costly for the solver and allows by filtering to improve the solving process.

The line 14 of the current program implements the *Constraint 4*. The rule ensures to have, given a selected intermediate gene I, at least 1 selected input G topologically predecessor of I (`ancestor(G,I)`) using a choice rule `1{...}`.

Finally, line 15 aims to maximize the number of matches comprising cells involved in a pseudo-perturbation.

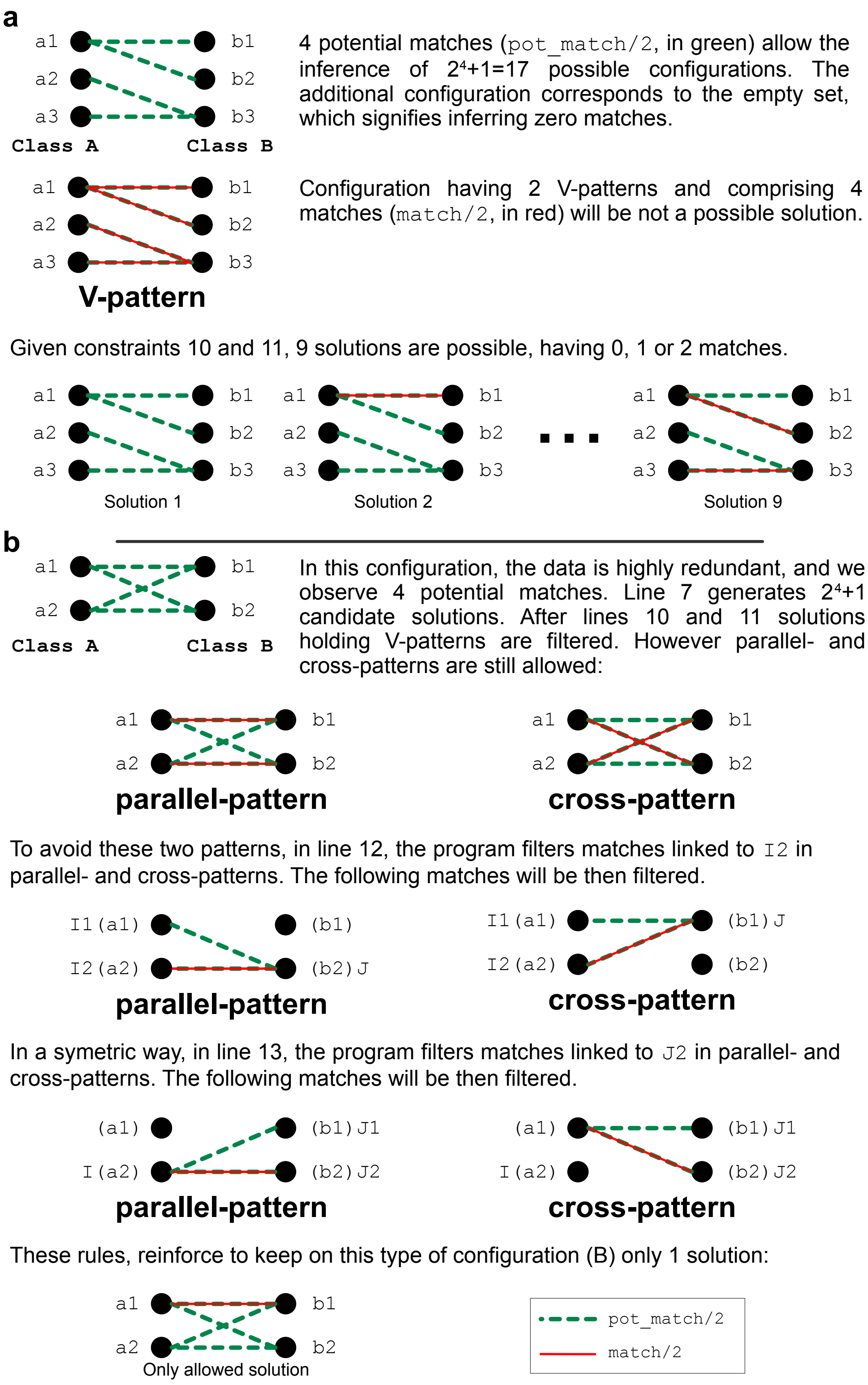

Supplementary Fig. 3 **Illustration of the *Constraint 2*.** The *Constraint 2* prevents redundancies in pseudo-perturbations through the lines 10 to 13 in ASP program. Diverse patterns contain redundancies and need to be forbidden. **a** Prohibition of V-patterns illustrated with a toy example. **b** Prohibition of parallel- and cross-patterns illustrated with a toy example.

13 Supplementary Note 6. Logical rules that make up the learned BNs.

Below, we list the logical rules found in the two families of BNs learned for Solution 1. We have merged all logical rules present in each BN within the family. These lists were generated from Caspo's output, which provides logical rules specific to each learned BN (networks.csv files; see the Zenodo repository, `TE_matureTE_discrimination` folder). The lists presented below are also available in the Zenodo repository for easier reuse (logical_rules.txt files).

TE logical rules

```
TERT<-!JUN
JUN/FOS<-FOS
TCF4<-JUN/FOS
DDIT3<-!MYC
E2F1<-!C21orf33
JUN<-SMAD3/SMAD4/JUN/FOS
ATF2/JUN<-JUN
PSAT1<-SMARCE1
FOS<-ELK1
SMAD3/SMAD4/JUN/FOS<-FOS
ATF3<-ATF2/JUN
C21orf33<-ATF2/JUN
JUN/JUND<-JUN
MYB<-E2F1+JUN/JUND
MYC/Max<-MYC
CEBPB<-MYB
CEBPB<-!MYC/Max
MYC<-!PCBP4+TERT
MYC<-!PCBP4+!TCF4
GSR<-JUN/FOS
```

For explanations, let's translate the two last rules. `MYC<-!PCBP4+!TCF4` means $MYC = \neg PCBP4 \wedge \neg TCF4$, and `GSR<-JUN/FOS` means $GSR = JUN/FOS$.

Mature TE logical rules

```
JUN/FOS<-FOS
DDIT3<-!MYC/Max/MIZ-1
DDIT3<-!MYC
JUN<-SMAD3/SMAD4/JUN/FOS
ATF2/JUN<-JUN
PSAT1<-SMARCE1
FOS<-ELK1
SMAD3/SMAD4/JUN/FOS<-FOS
MYC/Max/MIZ-1<-MYC
ATF3<-ATF2/JUN
MYC/Max<-MYC
CEBPD<-!MYC/Max/MIZ-1
CEBPB<-!MYC/Max
MYC<-JUN+!SKI
GSR<-JUN/FOS
```

References

1. Chebouba, L., Miannay, B., Boughaci, D. & Guziolowski, C. Discriminate the response of Acute Myeloid Leukemia patients to treatment by using proteomics data and Answer Set Programming. *BMC Bioinformatics* **19,** 15–26 (2018).
2. Bolteau, M., Bourdon, J., David, L. & Guziolowski, C. *Inferring Boolean Networks from Single-Cell Human Embryo Datasets* en. in *Bioinformatics Research and Applications* (eds Guo, X., Mangul, S., Patterson, M. & Zelikovsky, A.) (Springer Nature, Singapore, 2023), 431–441. ISBN: 9789819970742.
3. Moignard, V. *et al.* Decoding the regulatory network of early blood development from single-cell gene expression measurements. en. *Nature Biotechnology* **33,** 269–276. ISSN: 1546-1696. `https://www.nature.com/articles/nbt.3154` (2024) (Mar. 2015).
4. Dunn, S.-J., Martello, G., Yordanov, B., Emmott, S. & Smith, A. G. Defining an essential transcription factor program for naïve pluripotency. *Science* **344,** 1156–1160. `https://www.science.org/doi/10.1126/science.1248882` (2024) (June 2014).
5. Bonnaffoux, A. *et al.* WASABI: a dynamic iterative framework for gene regulatory network inference. en. *BMC Bioinformatics* **20,** 220. ISSN: 1471-2105. `https://doi.org/10.1186/s12859-019-2798-1` (2024) (May 2019).
6. Chevalier, S., Froidevaux, C., Pauleve, L. & Zinovyev, A. Synthesis of boolean networks from biological dynamical constraints using answer-set programming. *Proceedings - International Conference on Tools with Artificial Intelligence, ICTAI* **2019-November,** 34–41. ISSN: 9781728137988 (2019).
7. Gerstein, M., Kundaje, A., Hariharan, M., *et al.* Architecture of the human regulatory network derived from ENCODE data. *Nature* **489,** 91–100 (2012).
8. Meistermann, D. *Modélisation du développement préimplantatoire humain à partir de données de transcriptome de cellule unique* 2020NANT1019. PhD thesis (2020). `http://www.theses.fr/2020NANT1019/document`.
